# Macrophage metabolic reprogramming presents a therapeutic target in lupus nephritis

**DOI:** 10.1073/pnas.2000943117

**Published:** 2020-06-15

**Authors:** Chenzhi Jing, Tomas Castro-Dopico, Nathan Richoz, Zewen K. Tuong, John R. Ferdinand, Laurence S. C. Lok, Kevin W. Loudon, Gemma D. Banham, Rebeccah J. Mathews, Zaeem Cader, Susan Fitzpatrick, Kathleen R. Bashant, Mariana J. Kaplan, Arthur Kaser, Randall S. Johnson, Michael P. Murphy, Richard M. Siegel, Menna R. Clatworthy

**Affiliations:** ^a^Molecular Immunity Unit, Department of Medicine, Medical Research Council Laboratory of Molecular Biology, University of Cambridge, Cambridge CB2 0QH, United Kingdom;; ^b^National Institute of Arthritis and Musculoskeletal and Skin Disease, National Institutes of Health, Bethesda, MD 20892;; ^c^Division of Gastroenterology and Hepatology, Department of Medicine, University of Cambridge, Cambridge CB2 0QQ, United Kingdom;; ^d^Department of Physiology, Development, and Neuroscience, University of Cambridge, Cambridge CB2 3EG, United Kingdom;; ^e^Medical Research Council Mitochondrial Biology Unit, University of Cambridge, Cambridge CB2 0XY, United Kingdom;; ^f^National Institute of Health Research Cambridge Biomedical Research Centre, Cambridge CB2 0QQ, United Kingdom;; ^g^Cellular Genetics, Wellcome Sanger Institute, Hinxton CB10 1SA, United Kingdom

**Keywords:** Fcγ receptors, metabolism, lupus nephritis

## Abstract

IgG antibodies cause inflammation and organ damage in autoimmune diseases such as systemic lupus erythematosus (SLE). We investigated the metabolic profile of macrophages isolated from inflamed tissues in immune complex (IC)-associated diseases, including SLE and rheumatoid arthritis, and following IgG Fcγ receptor cross-linking. We found that human and mouse macrophages undergo a switch to glycolysis in response to IgG IC stimulation, mirroring macrophage metabolic changes in inflamed tissue in vivo. This metabolic reprogramming was required to generate a number of proinflammatory mediators, including IL-1β, and was dependent on mTOR and hypoxia-inducible factor (HIF)1α. Inhibition of glycolysis, or genetic depletion of HIF1α, attenuated IgG IC-induced activation of macrophages in vitro, including primary human kidney macrophages. In vivo, glycolysis inhibition led to a reduction in kidney macrophage IL-1β and reduced neutrophil recruitment in a murine model of antibody-mediated nephritis. Together, our data reveal the molecular mechanisms underpinning FcγR-mediated metabolic reprogramming in macrophages and suggest a therapeutic strategy for autoantibody-induced inflammation, including lupus nephritis.

## Significance

IgG antibodies are a key component of adaptive humoral immunity but can cause organ damage if they bind self-antigen, as occurs in the autoimmune disease systemic lupus erythematosus (SLE). Many of the proinflammatory effects of IgG are mediated by ligating Fcγ receptors (FcγRs) expressed by tissue-resident leukocytes such as macrophages. One of the most serious complications of SLE is kidney inflammation: lupus nephritis. Here we show that IgG ligation of FcγRs on macrophages in the kidney leads to a change in their metabolism, resulting in a switch toward glycolysis. Administration of a glycolysis inhibitor attenuated IgG-associated kidney macrophage activation, proinflammatory cytokine secretion, and kidney inflammation. Therefore, manipulating macrophage metabolism may be a useful therapeutic strategy in lupus nephritis.

IgG antibodies play an important role in defense against infection, but can cause inflammation and organ damage in autoimmune diseases such as systemic lupus erythematosus (SLE) (1). Patients with SLE have circulating antibodies that bind to a variety of self-antigens, resulting in IgG immune complex (IC) deposition in skin, joints, and kidneys, causing organ damage by activating complement and local immune cells (2, 3). Current treatments for lupus and other autoantibody-mediated diseases do not adequately control disease activity and tissue damage, and are associated with significant side effects (4); therefore, the identification of new therapeutic targets is a major unmet clinical need. Fcγ receptors (FcγRs) bind IgG IC and are expressed by many immune cells, including tissue-resident macrophages (5, 6). Polymorphisms in *FCGR* genes are associated with increased susceptibility to SLE and other autoimmune diseases (7–9), confirming their importance in disease pathogenesis. FcγRs may be activating (in humans, FcγRIIA, IIIA, IIIB) or inhibitory (FcγRIIB), and the balance of these two inputs determines the activation threshold and the magnitude of the inflammatory response to IgG IC (1, 10). Macrophages are tissue-resident immune cells that can respond to local immune challenges and, when stimulated by IgG IC, produce cytokines such as IL-6, TNFα, and IL-1β, as well as inflammatory mediators including prostaglandins and reactive oxygen species (ROS) (6, 7, 10–12). Given their potent proinflammatory effects in tissues, macrophages are an obvious therapeutic target in antibody-mediated autoimmunity. Indeed, mice deficient in activating FcγRs (13) or with macrophage-specific overexpression of the inhibitory FcγRIIB show less severe autoantibody-induced nephritis (14). These data suggest that inhibition of FcγR-dependent macrophage activation may be a useful treatment strategy in lupus and in other autoimmune diseases where antibodies play a pathogenic role.

There has been a recent appreciation that immune cells undergo metabolic reprogramming in response to local pathogen-derived signals and cytokines. Indeed, these changes in cellular metabolism can profoundly influence the nature of the immune response produced (15, 16). For example, macrophages activated by the toll-like receptor (TLR)-4 ligand lipopolysaccharide (LPS), known as M(LPS) or M1 macrophages (17), undergo an increase in glycolysis but a reduction in Krebs cycle-associated oxidative phosphorylation (OXPHOS) and have a proinflammatory phenotype (18, 19), whereas macrophages generated by IL-4 stimulation [M(IL-4) or M2 macrophages (17)] retain high OXPHOS and have antiinflammatory properties (18, 19). To date, there has been little consideration of how FcγR cross-linking by IgG IC affects metabolic processes in macrophages, and this information is important for our understanding of the pathogenesis of diseases characterized by antibody-mediated inflammation. Of note, although immune complex stimulation results in the production of proinflammatory cytokines by macrophages (11, 12), the signaling cascade downstream of FcγR is distinct from TLR signaling, involving SYK, PI3K, and MAPK (1, 5). Indeed, the addition of IgG IC to LPS-stimulated macrophages can even attenuate inflammation (20). This raises the question of whether FcγR cross-linking on macrophages may have distinct and specific effects on macrophage metabolism.

Here we show that tissue macrophages in IC-associated disease exhibit a glycolytic transcriptional signature, which is shared with macrophages following IgG IC stimulation in vitro. In response to IgG IC stimulation, macrophages up-regulate glycolytic genes and undergo a switch to aerobic glycolysis. This metabolic reprogramming was required to generate a number of proinflammatory mediators and cytokines, suggesting that this pathway could be activated in antibody-mediated tissue inflammation in vivo, and potentially represents a useful therapeutic target. In keeping with this, inhibition of glycolysis attenuated IgG IC-induced IL-1β production by kidney macrophages in mice and humans and reduced neutrophil recruitment and inflammation in nephrotoxic nephritis. Together, our data reveal the cellular molecular mechanisms underpinning FcγR-mediated metabolic reprogramming in macrophages and that this switch occurs in kidney macrophages in vivo following IgG IC challenge. Inhibition of macrophage glycolysis ameliorated autoantibody-induced inflammation, with therapeutic implications for conditions such as lupus nephritis.

## Results

### FcγR Cross-Linking Induces a Transcriptional Glycolytic Switch in Macrophages.

**Fig. 1.**
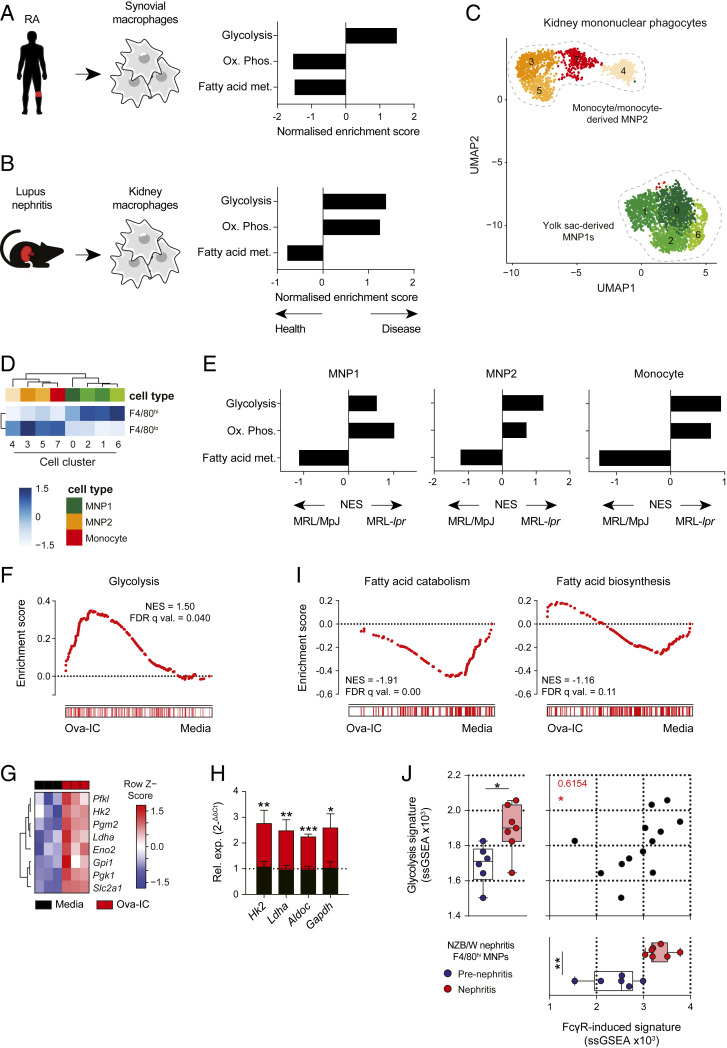
FcγR cross-linking induces a glycolytic transcriptional switch in macrophages. (*A* and *B*) Gene set enrichment analysis (GSEA) of selected Hallmarks metabolic pathways in synovial macrophages from RA patients (*A*) and renal macrophages from NZB/W mice (*B*). Data derived from GEO: GSE10500 and GSE27045, respectively. (*C*) UMAP plot of 3,353 single MNPs and associated cluster identities. (*D*) AUCell was used to test for enrichment of F4/80^hi^ or F4/80^lo^ gene signature (21), presented as a heat map showing the row-scaled mean enrichment score. Increasing color gradient indicates strength of enrichment (white to blue). (*E*) GSEA of select Hallmarks metabolic pathways in renal MNP subsets from *C* in nephritic MRL-*lpr* and control MRL/MpJ mice. (*F*) GSEA for Hallmarks glycolysis pathway in BMDMs stimulated with Ova-IC for 14 h. (*G*) Heat map of selected glycolysis genes from BMDMs shown in *F*. (*H*) qPCR analysis of selected glycolysis genes in murine BMDMs stimulated with Ova or Ova-IC for 6 h. (*I*) Fatty acid catabolism (GO: 0009062) and fatty acid biosynthesis (GO: 0006633) gene enrichment in BMDMs in *F*. (*J*) Correlation analysis of single sample (ss) GSEA scores for Hallmarks glycolysis pathway versus top 200 IC-induced BMDM genes in renal macrophages from *B*. Means ± SEM are shown for triplicate measurements and are representative of three independent experiments. *P* values were calculated using the two-tailed Student’s *t* test (*H*), nonparametric Mann–Whitney *U* test (*J*), and Spearman’s correlation (*J*; *P < 0.05; **P < 0.01, ***P < 0.001; ****P < 0.0001).

To address the question of whether inflammation associated with autoantibody IC deposition in tissues results in changes in macrophage metabolism, we assessed the transcriptional profiles of macrophages obtained from inflamed tissues. In human synovial macrophages isolated from patients with RA (Fig. 1*A*), and in kidney F4/80^+^ macrophages from mice with NZB/W lupus nephritis (Fig. 1*B* and *SI Appendix*, Fig. S1*A*), we observed an enrichment of glycolysis pathway genes compared to control macrophages. Kidney macrophages may arise from yolk-sac precursors or may be monocyte-derived and are F4/80^hi^CD11b^int^ and F4/80^int^CD11b^hi^, respectively (21), and may differ in their functional characteristics (22). To determine if the metabolic profile of both macrophage subsets was altered in IC-mediated inflammation, we performed single-cell RNA sequencing (scRNAseq) on renal myeloid cells sorted from a second model of murine nephritis, MRL-*lpr* mice, and from control MRL/MpJ mice. Several clusters of cells could be distinguished, with two major groups evident: mononuclear phagocyte (MNP) 1, with transcriptional similarity to yolk sac-derived F4/80^hi^ macrophages, and MNP2 that were transcriptionally similar to monocyte-derived macrophages and included a monocyte cluster (Fig. 1 *C* and *D*). In nondiseased MRL/MpJ kidney monocytes and macrophages, fatty acid metabolism genes were enriched (Fig. 1*E*). In contrast, in MRL-*lpr* mice, glycolysis and OXPHOS genes were increased in kidney MNPs, with glycolysis genes particularly enriched in monocyte-derived macrophages (Fig. 1*E*).

Although informative of potential metabolic changes induced by IgG, macrophages isolated from inflamed tissues may be influenced by a variety of tissue- and disease-specific factors, including local cytokines and danger-associated molecular patterns, as well as the exact nature of the IgG immune complexes. Furthermore, transcriptional changes in metabolic pathway genes require validation to definitively confirm cellular metabolic adaptations. To better characterize the specific effect of isolated FcγR cross-linking by IgG IC on macrophage metabolism, we stimulated murine bone marrow-derived macrophages (BMDMs) with a model IgG IC [ovalbumin opsonized with IgG (Ova-IC)] (23, 24) and assessed gene expression (*SI Appendix*, Fig. S1*B*). We observed a significant enrichment of glycolysis-associated genes following FcγR cross-linking (Fig. 1*F* and *SI Appendix*, Fig. S1*B*), including increased transcripts of key enzymes and transporters required for glycolysis, such as *Hk2*, *Ldha*, and *Slc2a1* (Fig. 1*G*), which we confirmed using real-time quantitative (q) PCR (Fig. 1*H*). Similarly, in human monocytes stimulated with plate-coated IgG (c-IgG), we also observed an enrichment of glycolysis-associated genes (*SI Appendix*, Fig. S1*C*). In addition to an increase in glycolysis genes, we also observed reduced expression of genes associated with fatty acid metabolism in IgG IC disease-associated macrophages and in murine macrophages following FcγR cross-linking (Fig. 1 *A*, *B*, and *E*), specifically fatty acid catabolism pathway genes (Fig. 1*I*). In vivo, inflamed NZB/W renal and RA synovial macrophages showed globally similar transcriptional changes to BMDMs stimulated with IgG IC, with an increase in FcγR-inducible genes and a reduction in FcγR-suppressed genes in BMDMs, suggesting that FcγR signaling may underpin the macrophage metabolic phenotype in vivo (*SI Appendix*, Fig. S1*D*). Furthermore, we observed a positive correlation between the induction of glycolysis pathway genes and the expression of the in vitro BMDM-derived FcγR-associated gene signature in NZB/W renal macrophages (Fig. 1*J*), supporting the conclusion that these pathways are causally linked.

Analysis of FcγR expression in renal macrophages from nephritic NZB/W mice demonstrated an increase in activating FcγR expression and reduction in FcγRIIB (*SI Appendix*, Fig. S1*E*), resulting in an increase in FcγR A:I ratio compared to prenephritic mice or mice in remission (*SI Appendix*, Fig. S1*F*). Therefore, inflamed tissue macrophages are primed for IgG ligation and exhibit an activated FcγR-associated transcriptional signature, including a switch to glycolysis. To ensure that this was not due to contamination of IgG IC with a Toll-like receptor (TLR) ligand such as lipopolysaccharide (LPS), we assessed glycolysis-associated genes in TLR2/4-deficient BMDMs and observed a similar increase in *HK2*, *Ldha*, *Aldoc*, and *Gapdh* expression post-FcγR cross-linking that was absent with LPS stimulation (*SI Appendix*, Fig. S1*G*). Together these data suggest that FcγR cross-linking by autoantibody-containing IgG IC initiates metabolic reprogramming in tissue macrophages toward glycolysis, with the potential to promote proinflammatory activity.

### FcγR Cross-Linking in Macrophages Results in a Switch to Aerobic Glycolysis.

**Fig. 2.**
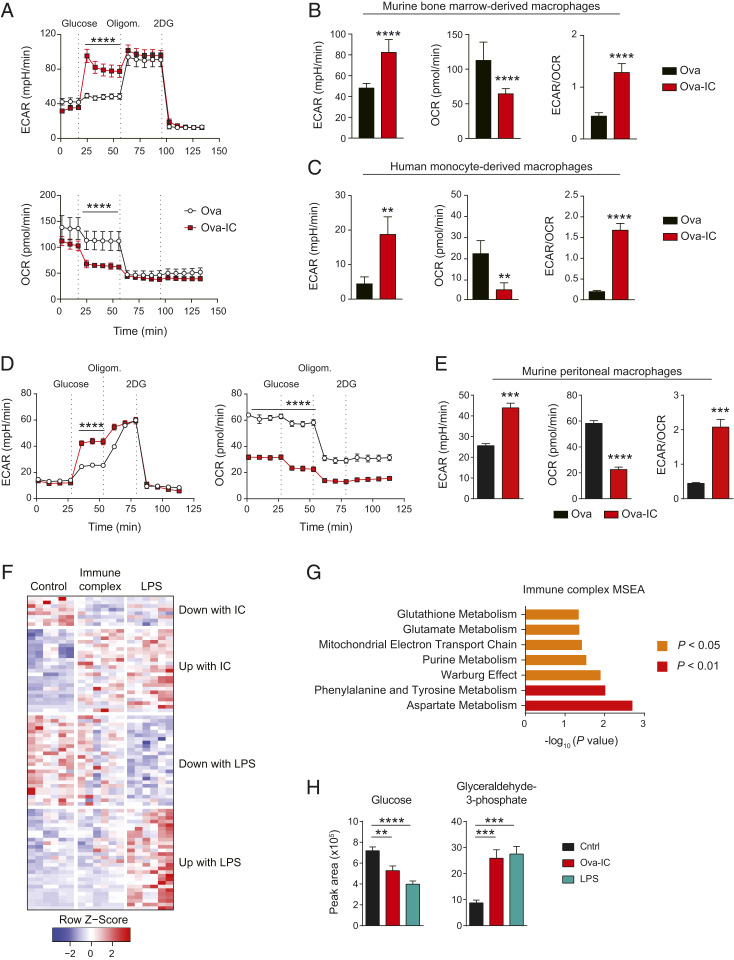
FcγR cross-linking in macrophages results in a switch to aerobic glycolysis. (*A*) ECAR and OCR in murine BMDMs stimulated with Ova or Ova-IC for 12 h were measured with a glycolysis stress test kit. (*B* and *C*) Quantification of ECAR, OCR, and ECAR/OCR ratio in Ova- or Ova-IC–treated murine BMDMs (*B*) and human MDMs (*C*) in the presence of glucose. Means ± SEM are shown, and data are representative of three independent experiments. (*D* and *E*) ECAR and OCR traces (*D*) and ECAR, OCR, and ECAR/OCR measurements in the presence of glucose (*E*) for murine peritoneal macrophages stimulated as in *A*. Mean ± SEM are shown, and data are representative of two independent experiments (*n* = 6 to 10 per group). (*F*) Heat map of differential metabolites in BMDMs stimulated with Ova (control), Ova-IC (immune complex), or LPS for 6 h. (*G*) Metabolite set enrichment analysis (MSEA) of differential metabolites in Ova-IC versus control macrophages. (*H*) Peak areas determined by mass spectrometry for glycolysis pathway metabolites altered by IgG IC stimulation in BMDMs stimulated as in *F*. *P* values were calculated using a two-way ANOVA (*A* and *D*), two-tailed Student’s *t* test (*B*, *C*, *E*, and *H*), and MSEA (*G*; *P < 0.05; **P < 0.01, ***P < 0.001; ****P < 0.0001).

To obtain a more detailed metabolic profile of macrophages following FcγR cross-linking and to validate our transcriptional analyses, we measured their extracellular acidification rate (ECAR) and oxygen consumption rate (OCR). We found an increase in ECAR and a decrease in OCR following IgG IC stimulation in both murine BMDMs (Fig. 2 *A* and *B*) and human monocyte-derived macrophages (Fig. 2*C*). Overall, the ECAR/OCR ratio was significantly increased following FcγR cross-linking (Fig. 2 *B* and *C*), demonstrating a switch to glycolysis. We also observed a similar metabolic switch in human monocyte-derived macrophages (MDMs) using an alternative model of IgG IC stimulation: IgG–anti-IgG Fab immune complexes (*SI Appendix*, Fig. S2*A*). To determine whether these observations are representative of tissue macrophages, we also performed ECAR and OCR measurements in murine peritoneal macrophages (*SI Appendix*, Fig. S2*B*). Following Ova-IC stimulation, we similarly observed a switch to glycolytic metabolism, with an increase in ECAR, a reduction in OCR, and elevated ECAR/OCR ratio (Fig. 2 *D* and *E*).

We next performed global metabolomic profiling of IgG IC-stimulated murine macrophages (using liquid chromatography–mass spectrometry) and compared these profiles to control or LPS-stimulated macrophages, the latter well-described to induce a switch to aerobic glycolysis. IgG-stimulated macrophages exhibited a unique metabolic profile compared with LPS-stimulated macrophages (Fig. 2*F* and *SI Appendix*, Fig. S2*C*), with specific increases in fumarate, inosine monophosphate (IMP), carbamoyl-aspartate (ureidosuccinic acid), and gamma-glutamylcysteine relative to control or LPS-stimulated macrophages (*SI Appendix*, Fig. S2 *C* and *D*). Notably, fumarate, IMP, and carbamoyl-aspartate are intermediates in aspartate metabolism. Indeed, metabolite set enrichment analysis (MSEA) demonstrated an enrichment in aspartate, and phenylalanine and tyrosine metabolism pathways, as well as metabolites associated with the Warburg effect in IC-stimulated macrophages (Fig. 2*G*), an MSEA profile that was distinct from that observed in LPS-stimulated macrophages (*SI Appendix*, Fig. S2*E*). In keeping with the MSEA and seahorse analysis, we observed a reduction in glucose and an increase in glycolysis intermediates, particularly glyceraldehyde 3 phosphate, in IgG IC-stimulated macrophages (Fig. 2*H* and *SI Appendix*, Fig. S2*C*). These data demonstrate that IgG ICs induce a change in macrophage metabolism, including the induction of aerobic glycolysis, with a metabolic phenotype that is overlapping with, but distinct from, that observed with LPS stimulation.

### IgG Immune Complex-Induced Glycolysis Is Required for Macrophage Production of IL-1β, PGE2, and ROS.

**Fig. 3.**
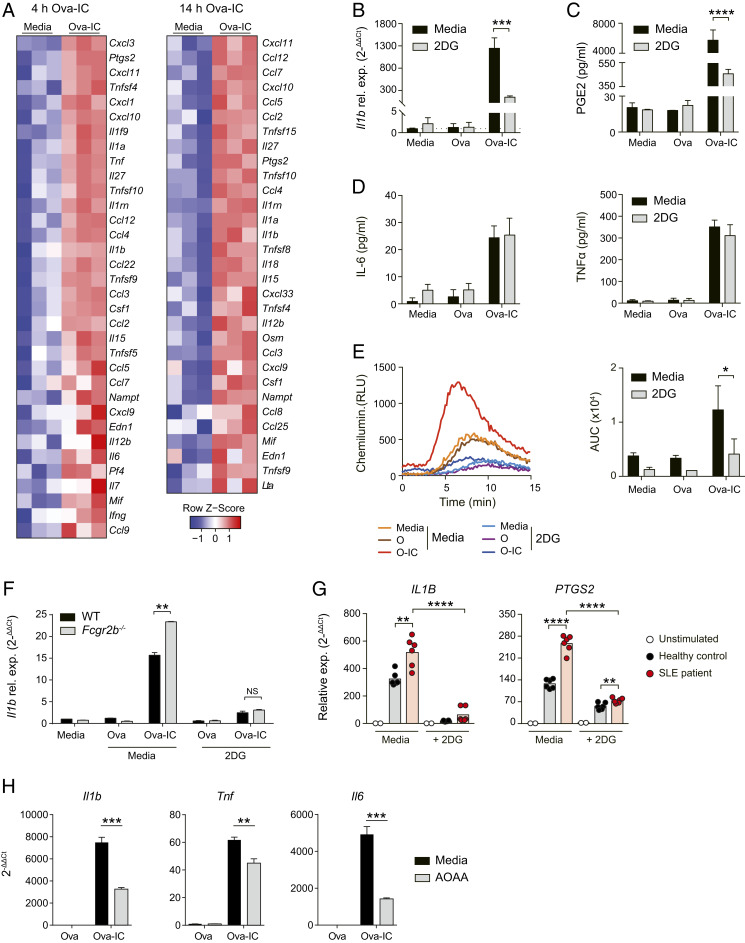
IgG-induced glycolysis is required for macrophage production of inflammatory mediators. (*A*) Transcriptomic analysis of differentially expressed (*P* < 0.05) cytokines and chemokines in BMDMs stimulated with Ova or Ova-IC for 4 h or 14 h. (*B*–*D*) Quantification of *Il1b* mRNA expression (*B*), PGE2 production (*C*), and IL-6 and TNFα production (*D*) by murine BMDMs stimulated with Ova/Ova-IC or unstimulated ± 2DG for 6 h. Means ± SEM are shown from triplicate measurements and are representative of three independent experiments. (*E*) Measurement of ROS production in murine BMDMs stimulated as in *B*–*D* for 2 h. Total production of ROS in each group was quantified by calculating the area under the curve (AUC; *Right*). Means ± SEM are shown from triplicate measurements and are representative of three independent experiments. (*F*) Quantification of *Il1b* mRNA production by BMDMs from WT or *Fcgr2b*^−/−^ mice stimulated as in *B*–*D*. Means ± SEM are shown from triplicate measurements and are representative of three independent experiments. (*G*) qPCR of *IL1B* and *PTGS2* mRNA in human MDMs treated with IgG-IC generated by incubating serum IgG from SLE patients or healthy controls with RNA/Sm antigen with or without the presence of 2DG for 6 h. Data are normalized to unstimulated controls (media) and *HPRT1*. (*H*) Quantification of cytokine expression in BMDMs stimulated with Ova/Ova-IC ± AOAA for 6 h. Means ± SEM are shown from four measurements and are representative of two independent experiments. *P* values were calculated using the two-tailed Student’s *t* test (*P < 0.05, **P < 0.01, ***P < 0.001, ****P < 0.0001).

FcγR cross-linking in BMDMs in vitro and tissue macrophages ex vivo induces the expression of several inflammatory cytokines and chemokines, including IL-1β, IL-6, and TNFα (Fig. 3*A* and *SI Appendix*, Fig. S3*A*). To determine whether the observed glycolytic switch impacted macrophage function and their capacity to induce inflammation, we stimulated BMDMs with IgG IC in the presence of 2-deoxy-D-glucose (2DG), an inhibitor of glycolysis. 2DG significantly attenuated IgG IC-induced IL-1β expression (Fig. 3*B* and *SI Appendix*, Fig. S3*B*) and PGE2 production (Fig. 3*C*), but had little impact on IL-6 and TNFα (Fig. 3*D*). IgG IC-induced ROS production was also inhibited by 2DG (Fig. 3*E*), while 2DG had no impact on FcγR-mediated phagocytosis of fluorescent Ova-IC (*SI Appendix*, Fig. S3*C*).

A single nucleotide polymorphism (SNP) in human *FCGR2B* (rs1050501) results in profound receptor dysfunction and is associated with increased susceptibility to lupus (7, 25). Similarly, *Fcgr2b*^−/−^ mice are prone to inducible and spontaneous antibody-mediated autoimmune disease and have exaggerated cellular responses to IgG IC (1, 10). We therefore assessed whether inhibition of glycolysis with 2DG might negate the heightened inflammatory response associated with FcγRIIB deficiency. We found that IL-1β induction by *Fcgr2b*^−/−^ BMDMs stimulated with IgG IC was restored to WT levels by the addition of 2DG (Fig. 3*F* and *SI Appendix*, Fig. S3*D*), suggesting that it may potentially ameliorate macrophage-induced inflammation in lupus. Indeed, to confirm the relevance of these observations to SLE in humans, we stimulated human monocyte-derived macrophages with autoantibody-containing IC generated from the serum of patients with SLE (26). This caused a significant increase in *IL1B* and *PTGS2* expression in macrophages that was attenuated by 2DG (Fig. 3*G*).

Given the alterations in aspartate metabolism (Fig. 2*G*), we also performed BMDM IgG IC stimulation in the presence of aminooxyacetate (AOAA), a broad-spectrum inhibitor of pyridoxal phosphate-dependent enzymes, including aspartate aminotransferase. Consistent with an important role of aspartate metabolism in FcγR-mediated inflammatory responses, we observed a reduction in Ova-IC–dependent inflammatory cytokine production, including *Il1b*, *Tnf*, and *Il6*, in the presence of AOAA (Fig. 3*H*).

### FcγR-Associated Glycolytic Switch Dependent on mTOR and HIF1α.

**Fig. 4.**
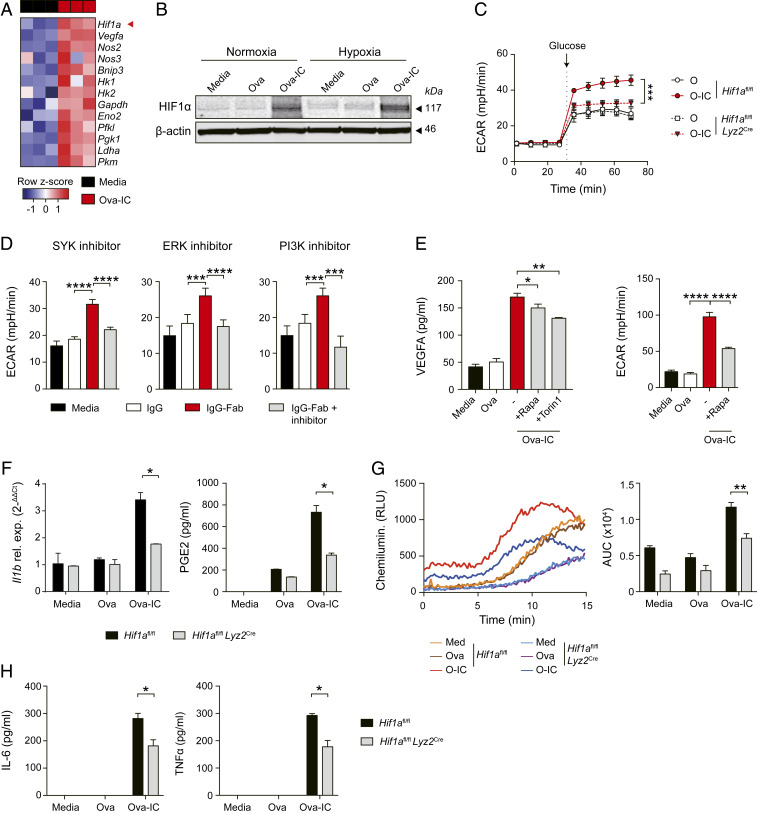
HIF1α activation modulates IC-induced glycolysis switch in macrophages. (*A*) Heat map showing transcriptomic analysis of *Hif1a* and HIF1α targets genes in murine BMDMs stimulated with Ova-IC or in controls (RPMI) for 14 h. (*B*) Western blot of HIF1α protein relative to β-actin in murine BMDMs ± Ova-IC stimulation under normoxic or hypoxic conditions. (*C*) HIF1α-deficient (*Hif1a*^*fl/fl*^ *Lyz2*^Cre^) and control (*Hif1a*^*fl/fl*^) BMDMs were stimulated with Ova or Ova-IC for 12 h. ECAR was measured before and after the addition of glucose. (*D*) ECAR was measured in control (media) or IgG- or IgG-Fab IC-stimulated human MDMs with the presence of small-molecule inhibitors. Macrophages were pretreated with SYK inhibitor (*Left*), ERK inhibitor (*Middle*), or PI3K inhibitor (*Right*) for 1 h and stimulated with IC (IgG-Fab) for 20 h. (*E*) Quantification of VEGFA in supernatants (*Left*) and ECAR (*Right*) from murine BMDMs stimulated with Ova, Ova-IC, or control (media) with or without the presence of mTOR inhibitors for 6 h. (*F*) Quantification of *Il1b* mRNA and PGE2 from HIF1α-deficient (*Hif1a*^fl/fl^ *Lyz2*^Cre^) and control (*Hif1a*^fl/fl^) BMDMs stimulated with Ova, Ova-IC, or control (media) for 6 h. (*G*) Measurement of ROS production in BMDMs stimulated as in *F* for 2 h (*Left*). Total production of ROS in each group was quantified by calculating the area under the curve (AUC; *Right*). (*H*) Quantification of IL-6 and TNFα from HIF1α-deficient (*Hif1a*^fl/fl^ *Lyz2*^Cre^) and control (*Hif1a*^fl/fl^) BMDMs stimulated as in *F*. All graphs show mean ± SEM from triplicate measurements and are representative of three independent experiments. ND, not detected. *P* values were calculated using a two-way ANOVA (*C*) or the two-tailed Student’s *t* test (*D*–*H*; *P < 0.05, **P < 0.01, ***P < 0.001, ****P < 0.0001).

Next, we sought to elucidate the molecular pathways underpinning IgG IC-induced metabolic reprogramming in macrophages. HIF1α is a transcription factor that can regulate the switch to glycolysis in macrophages stimulated with LPS, and is essential for some aspects of the inflammatory response (27–29). We found that exposure of BMDMs to IgG IC resulted in an increase in *Hif1a* transcripts and in the expression of several known HIF1α-target genes (Fig. 4*A*), as well as HIF1α protein (in both normoxic and hypoxic conditions; Fig. 4*B* and *SI Appendix*, Fig. S4*A*) and in VEGFA (*SI Appendix*, Fig. S4 *B* and *C*), an HIF1α-dependent gene that we have previously shown to be induced by IgG-FcγR signaling in subcapsular sinus macrophages in vivo (23). Furthermore, the increase in ECAR observed following FcγR cross-linking was significantly attenuated in HIF1α-deficient macrophages (Fig. 4*C*), demonstrating HIF1α-dependence.

To determine the molecular pathway involved in HIF1α activation in this context, we targeted known kinases downstream of activating FcγRs (*SI Appendix*, Fig. S4*D*). Following cross-linking by immune complexes, tyrosine phosphorylation of intracellular ITAMs leads to the activation of SYK-family kinases and downstream targets, including PI3K and ERK (5, 30–32). Small-molecule inhibitors of SYK, PI3K, and ERK attenuated IgG IC-mediated HIF1α activation (as evidenced by VEGFA secretion; *SI Appendix*, Fig. S4*E*) and the increase in ECAR in both murine (*SI Appendix*, Fig. S4*F*) and human macrophages (Fig. 4*D*), with ERK inhibition primarily impacting ECAR in human macrophages (Fig. 4*D* and *SI Appendix*, Fig. S4*F*). Since both PI3K and ERK can increase mammalian target of rapamycin (mTOR) activity by inhibiting TSC1/2 (*SI Appendix*, Fig. S4*D*), and mTOR mediates HIF1α induction in β-glycan–treated macrophages (33), we hypothesized that FcγR-mediated HIF1α activation might require mTOR. Consistent with this, mTOR inhibitors attenuated the increase in VEGFA and ECAR observed following the addition of IgG IC to murine (Fig. 4*E*) and human macrophages (*SI Appendix*, Fig. S4*G*). Together these data indicate that FcγR-induced glycolysis proceeds via an mTOR-HIF1α–dependent pathway.

To confirm the involvement of HIF1α in macrophage production of glycolysis-dependent inflammatory mediators following FcγR cross-linking, we stimulated HIF1α-deficient macrophages with IgG IC and observed a reduction in IL-1β expression and PGE2 and ROS production compared with control macrophages (Fig. 4 *F* and *G* and *SI Appendix*, Fig. S4*H*). However, in contrast to 2DG treatment, there was also an attenuation of IL-6 and TNFα in *Hif1a*^−/−^ macrophages (Fig. 4*H*), suggesting that the HIF1α-mediated increase in these cytokines is independent of its effects on glycolysis.

### Inhibiting Macrophage Glycolysis Reduces Immune Complex-Associated Neutrophil Recruitment In Vivo.

**Fig. 5.**
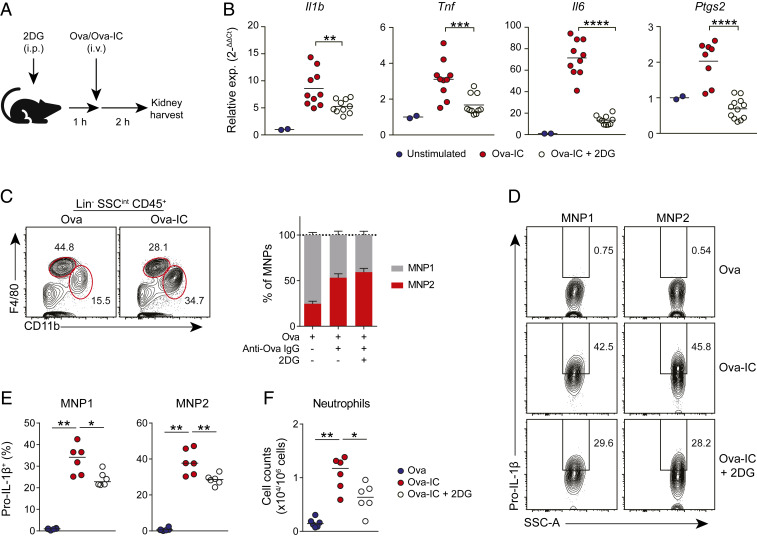
Inhibiting macrophage glycolysis reduces renal IL-1β and neutrophil recruitment in vivo. (*A*) Experiment design of the kidney-IgG IC model. (*B*) qPCR of *Il1b*, *Tnf*, *Il6*, and *Ptgs2* mRNA in whole kidney tissue of mice treated with Ova, Ova-IC, or Ova-IC + 2DG. Data are normalized to Ova controls and *Hprt*. (*C*) MNP1 and MNP2 gating and quantification in kidneys from mice treated as in *A* (*n* = 6 per group). Means ± SEM indicated. (*D* and *E*) Intracellular pro–IL-1β staining (*D*) and quantification (*E*) by flow cytometry for kidney MNP1 and MNP2 populations from mice treated as in *A* (*n* = 6 per group). Medians are indicated. (*F*) Quantification of CD11b^+^ Ly6C/G^hi^ neutrophils in mouse kidneys following Ova or Ova-IC injection with or without the pretreatment of 2DG (*n =* 6 per group). Medians are indicated. Data are representative of three independent experiments. *P* values were calculated using the nonparametric Mann–Whitney *U* test (*B*, *E*, and *F*; *P < 0.05, **P < 0.01, ***P < 0.001, ****P < 0.0001).

IL-1β is a proinflammatory cytokine with multiple functions in innate and adaptive immunity. One of its key effects is to augment inflammation by promoting neutrophil recruitment (34). We therefore investigated the effect of the IgG IC-mediated glycolytic switch in macrophages on IL-1β production and neutrophil recruitment in vivo. First, we used the peritoneal cavity as a model system, as described previously (35, 36) (*SI Appendix*, Fig. S5*A*). IgG-IC administered intraperitoneally was phagocytosed by peritoneal macrophages (*SI Appendix*, Fig. S5*B*). Although 2DG administration had no effect on peritoneal macrophage phagocytosis of IgG-IC (*SI Appendix*, Fig. S5*B*), it significantly decreased the magnitude of IgG IC-associated neutrophil recruitment (*SI Appendix*, Fig. S5*C*). To extend these observations to a tissue context more relevant to SLE, we assessed whether a glycolytic switch might occur in kidney-resident macrophages in response to circulating IgG IC (Fig. 5*A*). To do this, we treated mice with 2DG prior to i.v. injection of Ova–IgG immune complexes or free Ova. In mice treated with 2DG, we observed a reduction in IgG-IC–induced expression of *Il1b* and *Ptgs2*, as well as *Tnf* and *Il6*, in kidney tissue (Fig. 5*B*), demonstrating that 2DG is effective in suppressing IgG-induced inflammatory gene expression within the kidney. To investigate immune cell responses to circulating IgG-IC, we profiled kidney leukocytes by flow cytometry (*SI Appendix*, Fig. S5*D*). As noted previously, kidney macrophages can be broadly subdivided into two major populations: F4/80^hi^ yolk sac-derived macrophages [mononuclear phagocyte 1 (MNP1)] and CD11b^hi^ F4/80^int^ monocyte-derived macrophages (MNP2; *SI Appendix*, Fig. S5*D*). Following i.v. administration of IgG IC, there was an increase in monocyte-derived MNP2 in the kidney that was independent of glycolysis (Fig. 5*C*). Immune complex uptake was observed in both kidney macrophage populations, particularly MNP2 (*SI Appendix*, Fig. S5*E*), and 2DG treatment had no effect on immune complex phagocytosis (*SI Appendix*, Fig. S5*E*). Despite the variation in IgG-IC phagocytosis between kidney MNP populations, potentially due to differences in accessibility to i.v. IC or subsequent processing of internalized cargo (37), analysis of intracellular pro–IL-1β expression in kidney macrophage subsets demonstrated an increase in pro–IL-1β in response to circulating IgG-IC, which was inhibited by pretreatment with 2DG (Fig. 5 *D* and *E*). Consistent with the decrease in macrophage IL-1β, 2DG also attenuated IgG-IC induced neutrophil recruitment to the kidney (Fig. 5*F* and *SI Appendix*, Fig. S5*F*). In summary, targeting glycolysis is effective in suppressing pro–IL-1β expression by kidney macrophages and neutrophil recruitment in vivo, demonstrating the potential utility of this strategy to reduce antibody-mediated inflammation.

### Macrophage Glycolytic Switch as a Therapeutic Target in Immune Complex-Mediated Nephritis.

**Fig. 6.**
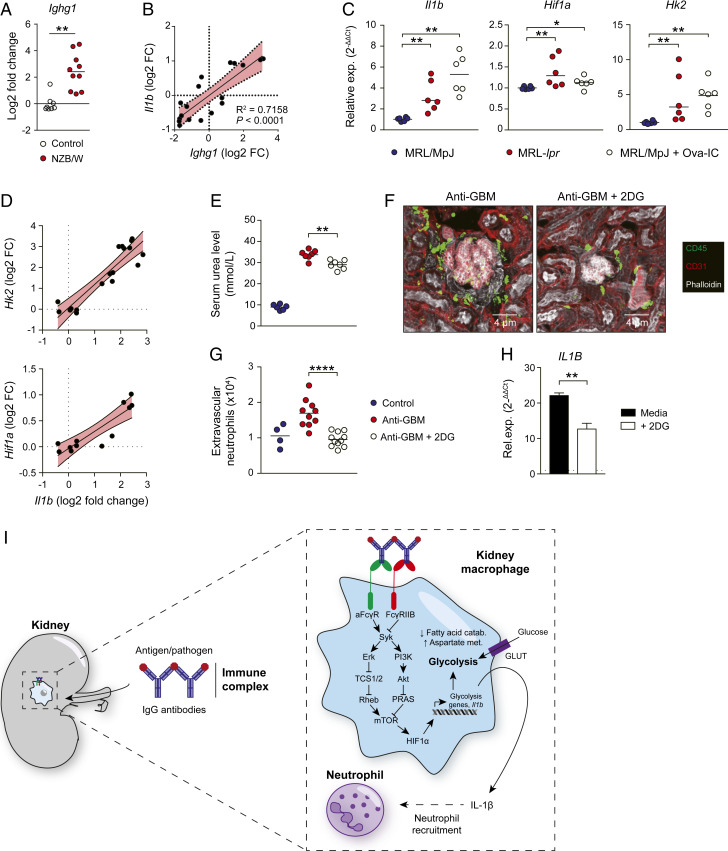
Inhibition of IC-induced glycolytic switch reduces autoantibody-mediated renal inflammation in vivo. (*A*) *Ighg1* expression in whole kidney tissue from NZB/W kidneys versus controls. Means are indicated. Data derived from GEO: GSE27045. (*B*) Correlation of *Il1b* with *Ighg1* mRNA levels in renal tissue from NZB/W mice shown in *A*. (*C*) qPCR of *Hk2*, *Hif1a*, and *Il1b* mRNA in renal tissue of MRL/MpJ and MRL-*lpr* mice and MRL/MpJ mice injected with Ova-IC. Data are normalized to gene expression in MRL/MpJ mice and *Hprt*. Means are indicated. (*D*) Correlation of glycolysis-associated genes and *Il1b* mRNA in renal tissue pooled from MRL/MpJ and MRL-*lpr* mice. (*E*) Serum urea levels in mice 24 h after i.v. injection of nephrotoxic serum (anti-GBM) with or without pretreatment with 2DG (*n* = 6 to 7 per group). Medians are indicated. (*F*) Representative kidney confocal images of mice treated as in *E*. (*G*) Quantification of neutrophils in kidneys of mice treated as in *G* (*n* = 4 to 10 per group). Medians indicated. (*H*) qPCR of human kidney cells stimulated with Ova-IC ± 2DG for 12 h. Data are normalized to Ova control and *HPRT1*. (*I*) Graphical summary of IgG IC-induced metabolic reprogramming in kidney macrophages. For in vivo experiments (*C*–*G*), medians are indicated, and each point represents a single kidney. For human kidney stimulations, means ± SEM are indicated from triplicate measurements. Data are representative of two or three independent experiment. *P* values were calculated using limma with multiple comparisons correction using the BH procedure (*A*), linear regression analysis (*B*), nonparametric Mann–Whitney *U* test (*C*, *E*, and *G*), or the two-tailed Student *t* test (*H*; *P < 0.05; **P < 0.01; ***P < 0.0001; ****P < 0.0001).

Renal involvement occurs in more than half of patients with SLE and is one of the most serious clinical manifestations of disease (3). In murine lupus nephritis, there was an increase in *Ighg1* expression within NZB/W kidney tissue, consistent with previous reports of local autoantibody production (38) (Fig. 6*A*), and a positive correlation between *Ighg1* transcripts and the expression of a number of nephritis-associated inflammatory mediators (*SI Appendix*, Fig. S6 *A* and *B*), including IL-1β (Fig. 6*B*), emphasizing the potential importance of glycolysis-associated IL-1β production in mediating autoantibody-associated inflammation in the kidney. To explore this further, we administered i.v. IgG IC to MRL/MpJ control mice. This increased renal *Hif1a* and *Il1b* transcripts to levels observed in diseased MRL-*lpr* kidneys (Fig. 6*C*). There was a significant positive correlation between *Hk2* and *Hif1a* and *Il1b* transcripts in MRL-*lpr* kidneys with lupus nephritis (Fig. 6*D*), implicating HIF1α-induced glycolysis in the induction of autoantibody-mediated inflammation in vivo. Of note, IL-1β has previously been identified in glomerular macrophages in diseased MRL-*lpr* mice (39), and our data reveal a potential molecular mechanism underpinning this observation.

To test whether inhibition of the macrophage glycolytic switch might represent a useful strategy to reduce kidney macrophage activation in nephritis, we treated mice with 2DG and then challenged them with nephrotoxic serum. Inhibition of glycolysis reduced serum urea levels (Fig. 6*E*) as well as leukocyte and neutrophil recruitment (Fig. 6 *F* and *G*). Finally, we sought to confirm our findings in primary human kidney macrophages using fresh kidney samples obtained from organ donors that had consented for research (22). As observed in monocyte-derived macrophages, treatment with 2DG decreased *IL1B* production following IC challenge (Fig. 6*H*), confirming the potential efficacy of this approach as a therapeutic strategy to reduce autoantibody-mediated inflammation in human kidneys.

## Discussion

It has been recently appreciated that macrophage stimulation with pathogen-derived danger signals and cytokines can lead to changes in metabolism that profoundly impact function (15), with LPS-stimulated M1 macrophages undergoing an increase in glycolysis and a reduction in Krebs cycle-associated OXPHOS and IL-4–stimulated M2 macrophages retaining high OXPHOS (18, 19). To date, the question of whether and how FcγR cross-linking by IgG IC might impact macrophage metabolism has been explored to only a limited extent (40). Our study confirmed a previous description that the metabolomics changes in IgG IC-stimulated macrophages are distinct from those observed in LPS-treated macrophages (40). However, we have gone on to show that, like TLR4 stimulation, IgG immune complexes trigger a switch away from OXPHOS toward glycolysis, with important functional effects. Notably, our transcriptomic analyses of macrophages isolated from IgG IC disease-associated tissues consistently showed an increase in glycolysis genes, but variable effects on OXPHOS genes, with a reduction in synovial macrophages in RA but an increase in kidney macrophages in lupus nephritis. Such differences are likely to reflect variability in organ-specific and disease-specific local stimuli, including cytokines, DAMPs, and nature of IgG (isotype and glycosylation). By studying macrophages in isolation following stimulation with only IgG IC, we confirmed the specific effects of FcγR cross-linking on macrophage metabolism, resulting in an increase in glycolysis and aspartate metabolism and a decrease in OXPHOS and fatty acid catabolism (Fig. 6*I*).

Our experiments demonstrated that IgG IC-induced IL-1β production was HIF1α- and glycolysis-dependent. IL-1β is a potent proinflammatory cytokine that has previously been identified in glomerular macrophages in diseased MRL-*lpr* mice (39) as well as *Fcgr2b*^−/−^ mice (41), while elevated renal IL-1 family cytokine responses are common to several models of nephritis (*SI Appendix*, Fig. S6*A*). Indeed, IL-1R1– or IL-1β–deficient mice are protected from anti-GBM IgG-mediated nephritis (42). Furthermore, it is noteworthy that *Fcgr2b*^−/−^ mice develop fatal glomerulonephritis that is dependent on IL-17 signaling (43), while renal Th17 cells are also observed in ANCA-associated glomerulonephritis in humans (44). Therefore, strategies aimed at suppressing IL-1β induction and downstream type 17 immune cell responses may show therapeutic potential in autoantibody-mediated renal inflammation.

Our data reveal the molecular mechanisms underpinning these observations and identify a pathway amenable to therapeutic intervention (Fig. 6*I*). Tissue macrophages differentiate in vivo and have significant transcriptional, phenotypic, and functional differences from monocyte- or bone marrow-derived macrophages and MNPs generated in vitro (45, 46). In our study, we utilized monocyte- and bone marrow-derived macrophages, but confirmed our findings in peritoneal macrophages and kidney-resident macrophages. Indeed, our use of primary human kidney macrophages, assayed ex vivo, provides evidence that the antiinflammatory effects of inhibiting macrophage glycolytic switch will be translatable to human nephritis.

Of note, a number of HIF1α inhibitors have been developed for clinical applications, mainly for the treatment of cancers, as malignant cells frequently up-regulate HIF1α (47). These drugs target HIF1α gene expression, protein stability, protein degradation, and DNA binding (48), and could be repurposed for the treatment of autoimmune inflammation. One caveat when considering this strategy is that many cells express HIF1α, leading to an unfavorable side-effect profile. However, protocols to target HIF1α inhibitors to tissue macrophages, for example, by conjugating to IgG so that they are taken up by phagocytic cells expressing FcγRs or by placing them in nanoparticles that localize to the kidneys (49), may overcome this limitation. Here we focused on acute models of nephritis, but future studies will be required to investigate the effect of longer-term HIF1α or glycolysis inhibition in chronic models of nephritis, such as in NZB/W mice, to determine the potential of these therapies for treating patients with lupus nephritis.

IgG antibodies are thought to drive inflammation in a number of autoimmune diseases beyond SLE, including rheumatoid arthritis, small vessel vasculitis, Sjogren’s syndrome, and systemic sclerosis (50–55). Our study raises the possibility that tissue-resident macrophages in joints, salivary glands, and skin may also be amenable to metabolic manipulation, as we have identified in kidney macrophages.

In conclusion, our data reveal that IgG stimulation of macrophages can profoundly alter cell metabolism via HIF1α and glycolysis induction. This metabolic switch occurred in kidney macrophages during antibody-mediated nephritis, and glycolysis inhibition attenuated tissue inflammation, highlighting its potential as a therapeutic strategy in lupus nephritis.

## Methods

### Mice.

Wild-type C57BL/6 mice were obtained from the Jackson Laboratories. *Hif1a*^fl/fl^ and *Lyz2*^Cre^ mice on a C57BL/6 background were obtained from the Jackson Laboratories and crossed to generate *Hif1α*^fl/fl^*Lyz2*^Cre^ mice. *Fcgr2b*^−/−^ mice on a C57BL/6 background were provided by J. Ravetch (Rockefeller University, New York, NY) and S. Bolland (National Institute of Allergy and Infectious Diseases, National Institutes of Health, Bethesda, MD). *Tlr2/4*^−/−^ mice were a gift from P. Tourlomousis (University of Cambridge, Cambridge, UK). MRL/MpJ (no. 00486) and MRL-*lpr* (no. 00485) mice were obtained from the Jackson Laboratories. In all experiments, both male and female mice were used. For all in vivo experiments, 6- to 12-wk-old mice were used. In the United Kingdom, mice were maintained in specific pathogen-free conditions at a Home Office-approved facility. All procedures were conducted in accordance with the United Kingdom Animals (Scientific Procedures) Act of 1986. In the United States, all animal study protocols were approved by the animal care and use committee (ACUC) of the National Institute of Arthritis and Musculoskeletal and Skin Diseases, listed on animal study protocol AO14-01–01, and in agreement with ARAC guidelines (3.18.1).

### Immune Complexes.

For ovalbumin immune complexes, endotoxin-free ovalbumin (no. 321000; Hyglos) was opsonized with a polyclonal rabbit anti-ovalbumin antibody (C6534, Sigma; 1:140, wt/wt) at 37 °C for 1 h. For in vitro phagocytosis assays, Alexa Fluor 647-conjugated ovalbumin was used (O34784; Thermo Fisher). For IgG immune complexes, human IgG (5172-9017; AbD Serotec)/mouse IgG (ab36355; Abcam) was opsonized with monoclonal human anti-human IgG antibody (HCA059; AbD Serotec)/goat F(ab′)2 anti-mouse IgG-(Fab′)2 antibody (ab98754; Abcam; 1:200, wt/wt) at 37 °C for 1 h. For in vivo experiments, 0.33 g/kg Alexa Fluor 647-conjugated ovalbumin (O34784; Thermo Fisher) was opsonized with 3.2 g/kg polyclonal rabbit anti-ovalbumin antibody (Sigma-Aldrich) at 37 °C for 1 h before injection. Details of systemic lupus erythematosus immune complexes are provided in the *SI Appendix*.

### Macrophage In Vitro Stimulation.

Details of the generation/isolation and culture of human MDMs, murine BMDMs, and murine peritoneal macrophages are provided in the *SI Appendix*. For extracellular flux analysis, macrophages were pretreated with ERK inhibitor (U0126; Sigma, 10 μM), PI3K inhibitor (wortmannin, no. 9951; Cell Signaling Technology, 1 μM), SYK inhibitor (Syk inhibitor IV; BAY61-3606, 1796–1,5; BioVision, 2 μM), mTOR inhibitor (rapamycin; Cell Signaling Technology, 10 nM; Torin1, no. 14379; Cell Signaling Technology, 1 μM), and AMPK activator (metformin, no. 13118; Cayman Chemical, 3 mM) for 1 h before the addition of IC. Cell culture supernatants were removed, and cells were washed using PBS before analysis. For cytokine production assay, BMDMs were treated with either antigens or corresponding IC (60 μg/mL) for 6 h. Cell culture supernatants were harvested and frozen at −20 °C until use. For glycolysis inhibition, macrophages were pretreated with 2-deoxy-D-glycose (5 mM, D8375; Sigma) for 45 min before adding the antigens or IC for 15 min to 6 h. For aspartate aminotransferase inhibition, BMDMs were treated with 5 mM aminooxyacetate (C13408; Sigma) prior to stimulation with Ova/Ova-IC for 6 h. For microarray, 2 × 10^6^ cells per well BMDMs were stimulated with either Ova (1 μg/mL) or Ova-IC (60 μg/mL) for 4 h or 14 h, supernatant removed, and cells lysed in the plate for RNA extraction.

### Extracellular Flux Analysis.

Macrophages were seeded in an XF96 microplate (Seahorse; Agilent Technology) at 75,000 cells per well. Cells were stimulated as described earlier for 20 h and washed/incubated with the Assay Medium [XF Base Medium (no. 102353; Seahorse; Agilent Technology), sodium pyruvate (Gibco, 2 mM), and L-glutamine (Gibco, 2 mM)] in a non-CO_2_ incubator at 37 °C for 1 h. Oxygen consumption rate and extracellular acidification rate were assessed with an XF96 Extracellular Flux Analyzer (Seahorse; Agilent). Glucose (Fisher Chemical, 10 mM), oligomycin (Sigma, 1 μM), and 2DG (Sigma, 100 mM) were injected to the plate sequentially. Data were analyzed using XF Wave software (version 2.3).

### In Vivo Kidney Macrophage Stimulation.

Wild-type C57BL/6 mice were first injected with 2DG (0.25 g/kg) or PBS (control) intraperitoneally. After 1 h, Alexa Fluor 647-conjugated immune complexes were injected via tail vein at a dose of 500 ng/g. FITC-conjugated anti-mouse CD45 monoclonal antibody (clone 30-F11; eBioscience; 75 μg/kg) was injected into mice i.v. after 2 h, immediately before mice were euthanized. Kidneys were collected and the visceral fat and kidney capsule removed. Kidneys were finely minced and digested in RPMI-1640 medium containing 10 mM Hepes, 32.5 μg/mL Liberase TM (Roche), and 0.1 mg/mL DNase I (Roche) for 25 min at room temperature. Tissue pieces were mechanically dissociated through a 70-μm cell strainer and washed with PBS containing 2% FBS, and red blood cell lysis was performed using distilled H_2_O containing 0.83% (wt/vol) NH_4_Cl, 0.1% (wt/vol) NaHCO_3_, 100 mM EDTA. Single-cell suspensions were subjected to a 44% (vol/vol) Percoll gradient (Sigma Aldrich) and washed thoroughly in ice-cold PBS prior to downstream analysis. A piece of tissue from each sample was also collected and stored in RNAlater stabilization solution (AM7020; Thermo Fisher) for the qPCR. Details of in vivo peritoneal macrophage stimulation are provided in the *SI Appendix*.

### Induction of Lupus Nephritis.

The anti-glomerular basement membrane (GBM) model (35, 36) was used to induce lupus nephritis in vivo. A total of 50 μL of sheep anti-rat GBM serum (PTX-001S; Probetex) was injected i.v. to wild-type C57BL/6 mice via the tail vein. The proteinuria level was monitored using Multistix 10 SG Reagent Strips (no. 03536597; Siemens). Mice were euthanized after 24 h, and kidneys were collected, processed, and analyzed by qPCR and flow cytometry as described earlier. For glycolysis inhibition, mice were pretreated with 2DG (0.25 g/kg), as described earlier, three times (−6 h, −3 h, −1 h) before the administration of anti-GBM.

### Human Kidney Derived Macrophages In Vitro Stimulation.

Cortex samples from human kidney were sliced into ∼30-mm^3^ pieces and digested for 30 min at 37 °C with agitation in a digestion solution containing 25 μg/mL Liberase TM (Roche) and 50 μg/mL DNase (Sigma) in RPMI-1640. Following incubation, samples were transferred to a gentle MACS C Tube (Miltenyi Biotec) and processed using a gentleMACS dissociator (Miltenyi Biotec) on program spleen 4 and subsequently lung 2. The resulting suspension was passed through a 70-μm cell strainer (Falcon) and washed with PBS before leukocyte enrichment using a Percoll density gradient (Sigma). Cells were counted using a hemocytometer with trypan blue. A total of 5 × 10^5^ cells per well were stimulated with Ova or Ova-IC with or without the presence of 2DG (5 mM/mL) for 12 h. Cells were then lysed and processed for RNA extraction and qPCR.

### Flow Cytometry.

Single-cell kidney and peritoneal suspensions were blocked with 0.5% heat-inactivated mouse serum, followed by extracellular staining for 1 h at 4 °C with a combination of antibodies listed in the *SI Appendix*. Viability staining was performed with Zombie UV Fixable Viability Kit (BioLegend) for 20 min at room temperature. For intracellular cytokine staining, cells were fixed and permeabilized using the Cytofix/Cytoperm kit (BD Bioscience) as per the manufacturer’s instruction. Staining was carried out for 1 h at room temperature using pro-IL-1β (NJTEN3; eBioscience) at a 1:200 dilution. Cell counting was performed using 123count eBeads (eBioscience). Flow cytometry data collection was performed on an LSR Fortessa flow cytometer (BD Biosciences), and data were analyzed using FlowJo software (Treestar, v10.2).

### Human Study Approval.

Human kidneys donated for transplantation but deemed unsuitable for implantation were used for in vitro stimulation experiments. All analysis of human material was performed in the United Kingdom. Ethical approval was granted by the local ethics committee (REC12/EE/0446), and the study was also approved by NHS Blood and Transplant. Serum from lupus patients was collected in the United States. Written informed consent was obtained from the healthy volunteers and from SLE patients. The enrollment of patients was approved by the National Institutes of Health Institutional Review Board (94-AR-0066).

### Data Availability.

The microarray data reported in this paper have been deposited in the Gene Expression Omnibus (GEO) database under the accession code GSE112081.

### Extended Methods.

Additional methodological details are provided in the *SI Appendix*.

## Supplementary Material

Supplementary File
