# Supplementary material for: Macrophage metabolic reprogramming presents a therapeutic target in lupus nephritis
Source: Proc Natl Acad Sci U S A. 2020 Jun 15;117(26):15160–71. doi: 10.1073/pnas.2000943117 (PMC7334513; doi:10.1073/pnas.2000943117)
Supplement: Supplementary File [file pnas.2000943117.sapp.pdf]

PNAS
www.pnas.org

Supplementary Information for

Macrophage metabolic reprogramming presents a therapeutic target in lupus nephritis

Chenzhi Jing[1#], Tomas Castro-Dopico[1#], Nathan Richoz[1,2], Zewen K. Tuong[1], John R. Ferdinand[1], Laurence S.C. Lok[1], Kevin W. Loudon[1], Gemma D. Banham[1], Rebeccah J. Mathews[1], Zaeem Cader[3], Susan Fitzpatrick[4], Kathleen R. Bashant[2], Mariana J. Kaplan[2], Arthur Kaser[3], Randall S. Johnson[4], Michael P. Murphy[5], Richard M. Siegel[2], Menna R. Clatworthy[1*]

Menna R. Clatworthy
Email: mrc38@cam.ac.uk

This PDF file includes:

Supplementary methods
Figures S1 to S6
SI references

www.pnas.org/cgi/doi/10.1073/pnas.2000943117

Supplementary methods

Macrophage culture. Murine bone marrow was flushed from femurs and tibias, plated in regular petri dishes and supplemented with 100 ng/ml macrophage colony-stimulating factor (M-CSF, 315-02, Peprotech) in complete medium (10 % FBS (11306060, Hyclone), 1X penicillin/streptomycin (10003927, Thermo Fisher) in RPMI 1640 (R8758, Sigma)) twice over 5 days. On day 6, macrophages were transferred to tissue culture plates for stimulation. Peritoneal macrophages were harvested by injection of 5 ml ice-cold sterile PBS into the peritoneal cavity. Peritoneal wash was plated in complete medium for 1 h in a 6-well plate and adherent cells were harvested for downstream experiments and re-plated in complete medium. Human monocyte-derived macrophages were generated from the peripheral blood of healthy volunteers. Peripheral blood mononuclear cells (PBMC) were isolated from leukocyte dense cones (NHS Blood and Transplant, Cambridge) through Ficoll-Hypaque density separation. Macrophages were generated by culturing purified PBMCs for 6 days with human M-CSF (100 ng/ml, 300-25, PeproTech) in complete medium and plated out at day 7 for subsequent stimulations.

Systemic lupus erythematosus immune complexes. Human anti-Sm/RNP immune complexes were prepared as described previously (1). Briefly, Serum from 3 SLE patients (All female, Ethnicity (Age): Hispanic (58); Hispanic (56); Asian (41)) and 3 healthy controls (All female, Ethnicity(Age): Hispanic (39); Asian (29); African American (50)) that had high Sm/RNP titers was purified with Melon Gel Monoclonal IgG Purification kit (45214, Thermo Fisher) following manufacturer's instruction. The purity of IgG was assessed by colloidal blue staining (LC6025, Thermo Fisher) and IgG concentration was measured by BSA assay (78426, Thermo Fisher). The purified IgG (10 μg/ml) was mixed with RNA/Sm antigen (10 μg/ml, Arotsc, ATR01-10) at a 1:1 ratio in RPMI and incubated at room temperature for 30 minutes. Human MDM were pre-treated with or without 2DG for 45 minutes, and IC was then added to the cells. After 6 hours, cell culture supernatant was collected, and cells were lysed for RNA extraction.

***In vivo* peritoneal macrophage stimulation.** Wild type C57BL/6 mice were injected with 2DG (0.25 g/kg) or PBS (control) intraperitoneally. After 1 h, Alexa Fluor 647-conjugated immune complexes were injected intraperitoneally at a dose of 0.5 g/kg. Mice were sacrificed 1 h later and peritoneal cavity resident immune cells were collected by injecting 5 ml of ice-cold PBS (with 3 % fetal bovine serum) into the peritoneal cavity. Peritoneal lavage was collected and cells were stained for flow cytometry analysis.

Reactive oxidative species assay. Kinetics of the oxidative burst was assessed using a horseradish peroxidase (HRP) – luminol – dependent chemiluminescence method. Macrophages were pre-treated with 2DG and then stimulated with Ova or Ova-IC as described above. Washed macrophages at a dilution of 10^7 cells per ml were warmed to 37 ºC for 15 min. 150 μl of macrophages were pre-incubated for 3 min with luminol (2 μM) and horseradish peroxidase (62.5 IU/ml) before being stimulated with PBS (control) or PMA (200 ng/ml, Sigma). PBS and PMA were added through the injection port. Light emission was assessed using a Berthold MicroLumat Plus luminometer (Berthold Technologies). Data output was in relative light units. Curves shown are constructed using three technical or biological replicates where indicated. Area under the curve (AUC) was calculated using GraphPad Prism (Version 7.0a).

RNA extraction and quantitative polymerase chain reaction. Tissue suspensions (homogenised using a Precellys Lysing Kit (MK28-R, Bertin Technologies)) or cultured macrophage RNA was extracted using a PureLink RNA Mini Kit (12183025, Thermo Fisher) according to the manufacturer's instructions. RNA concentration and purity were determined using a NanoDrop spectrophotometer (Thermo Fisher) prior to cDNA synthesis using a High Capacity RNA to cDNA Kit (4387406, Applied Biosystems). All qPCR was carried out in triplicate with Taqman reagents (Thermo Fisher) on the Viia7 qPCR system (Life Technologies). Primers for genes of interest (Taqman Gene Expression Assay, Life Technologies) are as follows: for mice, *Hk2* (Mm_00443385_m1), *Ldha* (Mm_01612132_g1), *Aldoc* (Mm_01298116_g1), *Gapdh*

(Mm_99999915_g1), *Il6* (Mm00446190_m1), *Tnf* (Mm00443258_m1), *Il1b* (Mm_00434228_m1), *Ptgs2* (Mm_00478374_m1), *Hif1a* (Mm00468869_m1), *Hprt* (Mm_03024075_m1); for human, *IL1B* (Hs_01555410_m1), *PTGS2* (Hs00153133_m1), *HPRT1* (Hs99999909_m1). Gene expression was normalised to *Hprt* using the $2^{-\Delta Ct}$. The comparative CT method ($2^{-\Delta\Delta Ct}$) was used for normalisation between experimental conditions and genotypes. For microarray, macrophages RNA was extracted using a PureLink RNA Mini Kit (12183025, Thermo Fisher) according to the manufacturer's instructions. Extracted RNA was washed three times with ethanol and spin-dried twice before being eluted into 50 µl RNAase-free H_2O.

Enzyme-linked immunosorbent assay. Concentrations of cytokines and VEGFA in cell supernatants were measured using ELISA Duoset kits (R&D Systems) for mouse TNFα (DY410), IL-6 (DY406), IL-1β (DY401) and VEGFA (DY493), according to the manufacturer's instruction. PGE2 concentration in cell supernatants was measured using the Monoclonal Prostaglandin E2 ELISA Kit (514010, Cayman Chemical). Optical density values were measured at a wavelength of 450 nm. Concentrations were calculated using an 8-parameter fit curve. The optical densities were measured at 450 nm using a CLARIOstar spectrophotometer (BMG Labtech). Data was analysed using the MARS Data Analysis Software (BGM Labtech, Version 5.01R2).

Flow cytometry. Combinations of the following anti-mouse antibodies were used: Gr1 (clone RB6-8C5, eBioscience), CD11b (clone M1/70, Tonbo Biosciences), CD11c (clone N418, BioLegend), F4/80 (clone BM8, BioLegend), CD45 (clone 30-F11, eBiosciences), CD3 (clone 17A2, BioLegend), CD19 (clone 6D5, BioLegend), MHCII (clone AF6-120.1, eBiosciences), CD45.2 (Clone 104, eBioscience). All antibodies were used at 1/200 dilution in PBS.

Immunofluorescence. Kidney tissues were fixed in 1 % paraformaldehyde (Electron Microscopy Services) / L-lysine / sodium periodate (both Sigma-Aldrich) buffer for 24 h followed by 8 h in 30 % sucrose in P-buffer. 30 mm sections were permeabilised and blocked in 0.1 M TRIS, containing 0.1 % Triton (Sigma), 1 % normal mouse serum, 1% BSA (R&D). Samples were stained for 16 h at 4°C in a wet chamber with the following antibodies: PE rat anti-mouse CD31 (1:50, clone MEC13.3, BD), Alexa Fluor 488 anti-mouse CD45 (1:200, clone 30-F11, BioLegend), Alexa Fluor 647 Phalloidin (1:100, Invitrogen). Samples were washed 3 times in PBS and mounted in Fluoromount-G (Southern Biotech). Images were acquired using a TCS SP8 (Leica, Milton Keynes, UK) confocal microscope. Raw imaging data were processed using Imaris (Bitplane).

Immunoblotting. Protein samples from cultured cells were prepared by direct lysis of cells in RIPA buffer (Sigma, R0278) with protease inhibitor (Sigma, P8340), followed by centrifugation at 14000 rpm for 10 min at 4ºC. The quantity of protein was measured using the Pierce BCA Protein Assay Kit (23227, Thermo Scientific). The supernatants containing 10 µg protein were heated at 95ºC for 5 min and resolved on Nupage 4-12 % Bis-tris Gel (Life Technologies) and were then transferred onto polyvinylidene difluoride (PVDF) membrane using iBlot Dry Blotting System (Life Technologies). Membranes were blocked in 5 % (w/v) dried milk (70166, Sigma) in Tris-Buffered Saline (TBS) for at least 1 h at room temperature and incubated with primary anti-HIF1α antibody (1:500, Ab51608, Abcam) overnight at 4ºC. Membranes were washed using milk/PBS-Tween and incubated with appropriate horseradish peroxidase-conjugated secondary antibody (Dylight conjugated anti-rabbit antibody (1:20000, Cell signalling, 5151S) at room temperature for 2 h. Loading control antibody used was anti-β-actin antibody (1:500, MA5-15739, Thermo Fisher). The blot was developed with the LICOR Odyssey CLx Imager (LI-COR Biosciences). Images were quantified using ImageJ software (1.49V) and normalised to loading control bands.

Extraction of aqueous metabolites. Following 6 h stimulation with Ova-IC or LPS, BMDMs were washed twice with PBS followed by addition of pre-chilled 4:1 methanol:water. Cells were scraped and the resulting methanol mixture transferred to 2 ml-flat-bottomed screw cap tubes. Samples were then vortexed, sonicated and lastly centrifuged at 21,000 *g* for 10 min to pellet any debris. The supernatant was transferred to new 2 ml tubes for drying and then subjected to further extraction using the methanol:chloroform method described by Folch (2). Briefly, a stainless steel ball (Qiagen) was added to each dried pellet on dry ice along with 1 ml of ice cold 2:1

chloroform:methanol inside a 2 ml-flat-bottomed screw cap tube (Starlab). The samples were homogenised using a Tissue Lyser (Qiagen) for 5 min at 25 Hz. 400 µl of ice-cold water was added and the samples thoroughly vortexed and sonicated for 5 min before centrifugation at 21,000 *g* for 5 min. After centrifugation the aqueous (top layer) fraction was separated and aliquoted into separate screw-cap tubes and kept on dry ice. A further 1 ml of 2:1 chloroform:methanol was added to the original tube containing the pellet and the extraction repeated as described above. The resulting aqueous fraction layers were combined, dried and stored at -20°C prior to further preparation and analysis. All solvents used were HPLC grade or higher and obtained from Honeywell (Fisher Scientific).

LC-MS sample preparation. Aqueous extracts of cells were lyophilised using a centrifugal evaporator (Savant, Thermo Scientific) and reconstituted in 100 µl of 7:3 acetonitrile: 0.1 M aqueous ammonium carbonate containing 2 µM [$^{13}C_{10}{}^{15}N_5$] adenosine monophosphate, [$^{13}C_{10}{}^{15}N_5$] adenosine triphosphate, 10 µM [$^{13}C_4$] succinic acid and 10 µM [$^{13}C_5{}^{15}N_5$] glutamic acid (all from Sigma Aldrich except the glutamic acid from Cambridge Isotope Laboratories) as internal standards. The resulting solution was vortexed then sonicated for 15 min followed by centrifugation at 21,000 *g* to pellet any remaining undissolved material. After centrifugation the supernatants were transferred with an automatic pipette into a 300 µl vial (Fisher Scientific) and capped ready for analysis. Ammonium carbonate and ammonium acetate were Optima™ grade obtained from Fisher Scientific.

LC-MS analysis of aqueous metabolites. For untargeted analysis, a Q Exactive Plus orbitrap coupled to a Vanquish Horizon ultra high performance liquid chromatography system was used. Samples were then analysed using a bridged ethylene hybrid (BEH) amide hydrophilic interaction liquid chromatography (HILIC) approach for the highly polar aqueous metabolites. For this analysis the strong mobile phase (A) was 100 mM ammonium carbonate, the weak mobile phase was acetonitrile (B) with 1:1 water:acetonitrile being used for the needle wash. The LC column used was the BEH amide column (150 × 2.1 mm, 1.7 µm, Waters). The following linear gradient was used: 20 % A in acetonitrile for 1.5 min followed by an increase to 60 % A over 2.5 min with a further 1 min at 60 % A after which the column was re-equilibrated for 1.9 min. After each chromatographic run the column was washed with 30 column volumes of 6:4 water:acetonitrile followed by a further 10 column volumes of 95:5 acetonitrile:water for storage. The total run time was 7 min, the flow rate was 0.6 ml/min and the injection volume was 5 µl. After HILIC analysis samples were dried and reconstituted in the same volume of 10 mM ammonium acetate prior to orthogonal mixed mode analysis using an ACE Excel C18-PFP column (150 × 2.1 mm, 2.0 µm, Hichrom). Mobile phase A consisted of water with 10 mM ammonium formate and 0.1 % formic acid and mobile phase B was acetonitrile with 0.1 % formic acid. For gradient elution mobile phase B was held at 0 % for 1.6 min followed by a linear gradient to 30 % B over 4.0 minutes, a further increase to 90 % over 1 min and a hold at 90 % B for 1 min with re-equilibration for 1.5 minutes giving a total run time of 6.5 minutes. The flow rate was 0.5 mL/min and the injection volume was 2 µl. The needle wash used was 1:1 water:acetonitrile.

For untargeted analysis using the Q Exactive Plus a full scan of 60-900 *m/z* was used at a resolution of 70,000 ppm where positive and negative ion mode assays were run separately in order to maximise data points across a peak at the chosen resolution. Source parameters used for the orbitrap were a vaporizer temperature of 400°C, an ion transfer tube temperature of 300°C, an ion spray voltage of 3.5 kV (2.5 kV for negative ion mode) and a sheath gas, auxiliary gas and sweep gas of 55, 15 and 3 arbitrary units respectively with an S-lens RF (radio frequency) of 60 %. For analysis of CoA species and reducing equivalents using the Q Exactive Plus orbitrap unique mass spectrometry methodology was employed where the full scan mass range was reduced to 500-1000 *m/z*, the capillary temperature was increased to 350°C and the S-lens RF to 100 %.

LC-MS data processing. For untargeted multivariate analysis, data were processed using Compound Discoverer (Version 2.1, Thermo Fisher Scientific) to determine unique LC-MS features with differential abundance between sample groups. For each differential MS feature, chromatogram peaks were manually verified using Xcalibur (Version 3.0, Thermo Fisher Scientific).

Accurate *m/z* values of putative compounds were compared against the METLIN database (Scripps Research Institute) including $[M+H]^+$, $[M+Na]^+$, $[M+NH_4]^+$ for positive mode and $[M-H]^-$, $[M+Cl]^-$ for negative mode ion adducts with a mass tolerance of 2ppm. A combination of MS/MS fragmentation profile, molecular formulae calculation based on isotope pattern and expected chromatographic chemical behaviour was then used to attribute metabolite identity. In case of ambiguity, external standards were used to confirm metabolite identification. Data from positive and negative ionisation modes were combined and duplicate metabolite identities removed. Data was normalised to total ion content and graphically depicted as a heatmap. Metabolite set enrichment analysis was performed using MetaboAnalyst 4.0 (McGill University, Canada) Enrichment Analysis tool.

Transcriptomic analysis. Previous published transcriptomic datasets (GSE10500, GSE27045, GSE107705, GSE102728, GSE109040) were obtained from the Gene Expression Omnibus (http://www.ncbi.nlm.nih.gov/geo/) along with appropriate chip annotation data. For investigation of the transcriptomic effect of immune complex stimulation on murine BMDMs we performed microarray analysis using the MoGene-2_1-st Affymetrix chip using the Gene Titan system (Affymetrix). Resulting data was uploaded to GEO under accession number GSE112081. Microarray data analysis and heatmap generation were conducted using GEO2R (http://www.ncbi.nlm.nih.gov/geo2r/) and Bioconductor (https://www.bioconductor.org) packages (Biobase, GEOquery, limma, Affy and gplots) in the R statistical environment. For RNA-seq, differential expression analysis was performed using DESeq 2 using a linear model with an appropriate design matrix following the default workflow. Gene Set Enrichment Analysis (GSEA, http://software.broadinstitute.org/gsea/) was conducted using the GSEA 4.0.1 according to the developers' instruction. Gene sets were downloaded from the Molecular Signature Database (MSigDB).

Single cell RNA sequencing. Single-cell RNAseq was performed on $CD11b^+F4/80^+$ mononuclear phagocytes (MNPs) sorted from *n* = 6 19-week-old MRL-*lpr* and *n* = 10 11-week-old MRL/MpJ mouse kidneys where each sorted sample was processed through the 3' Chromium kit (10x Genomics) as two individual channels where 10,000 cells were aimed to be recovered for each sample (performed at NIH facility) and sequenced on a HiSeq4000 (Illumina). Kidneys were processed as described in main Methods. The single-cell data (10x *cellranger* output) was processed using standard *Seurat*-inspired *scanpy* workflow (3, 4) with standard quality control steps; cells were filtered if number of genes > 2500 or < 200 and percentage mitochondrial content >= 5%. Genes were retained if is expressed by at least 3 cells. Doublet detection was performed using *scrublet* (5) with adaptations (6). Batch correction was performed using *bbknn* with samples (MRL/MpJ or MRL-*lpr*) as the batch term (7). Clustering was performed using Leiden algorithm (8). To group clusters into either resident MNP1 or infiltrating MNP2/monocytes, marker gene detection was performed using the Wilcoxon test implemented in *Seurat* and further gene set testing was performed using AUCell (9) using a murine kidney $F4/80^{hi}$ vs $F4/80^{lo}$ gene signature curated from E-MEXP-3510 (10). Pre-ranked GSEA was performed using *fgsea* (11) on a pre-ranked gene list defined using -log10(p-value)*sign of log2 fold-change from pair-wise differential gene test (Seurat, Wilcoxon test) performed between MRL/MpJ versus MR-*lpr* cells within each of the three cell type groups.

Statistics. Statistical analysis was preformed using Graphpad PRISM software (La Jolla, Version 7.0a). Unless otherwise stated data are expressed as mean ± SEM. For in vivo experiments, comparison between experimental groups was performed using a nonparametric Mann-Whitney-U test. A two-tail Student t-test was applied for in vitro stimulation experiments. For correlations of RNA expression levels, linear regression analysis was used. Outliers were identified using the ROUT method within Graphpad PRISM (Version 7.0a). All experiments were subject to at least three technical replicates per experimental parameter, and all data shown are representative of at least 3 individual experiments with 3 biological replicates, unless where otherwise indicated. * $P < 0.05$, ** $P < 0.01$, *** $P < 0.001$, **** $P < 0.0001$.

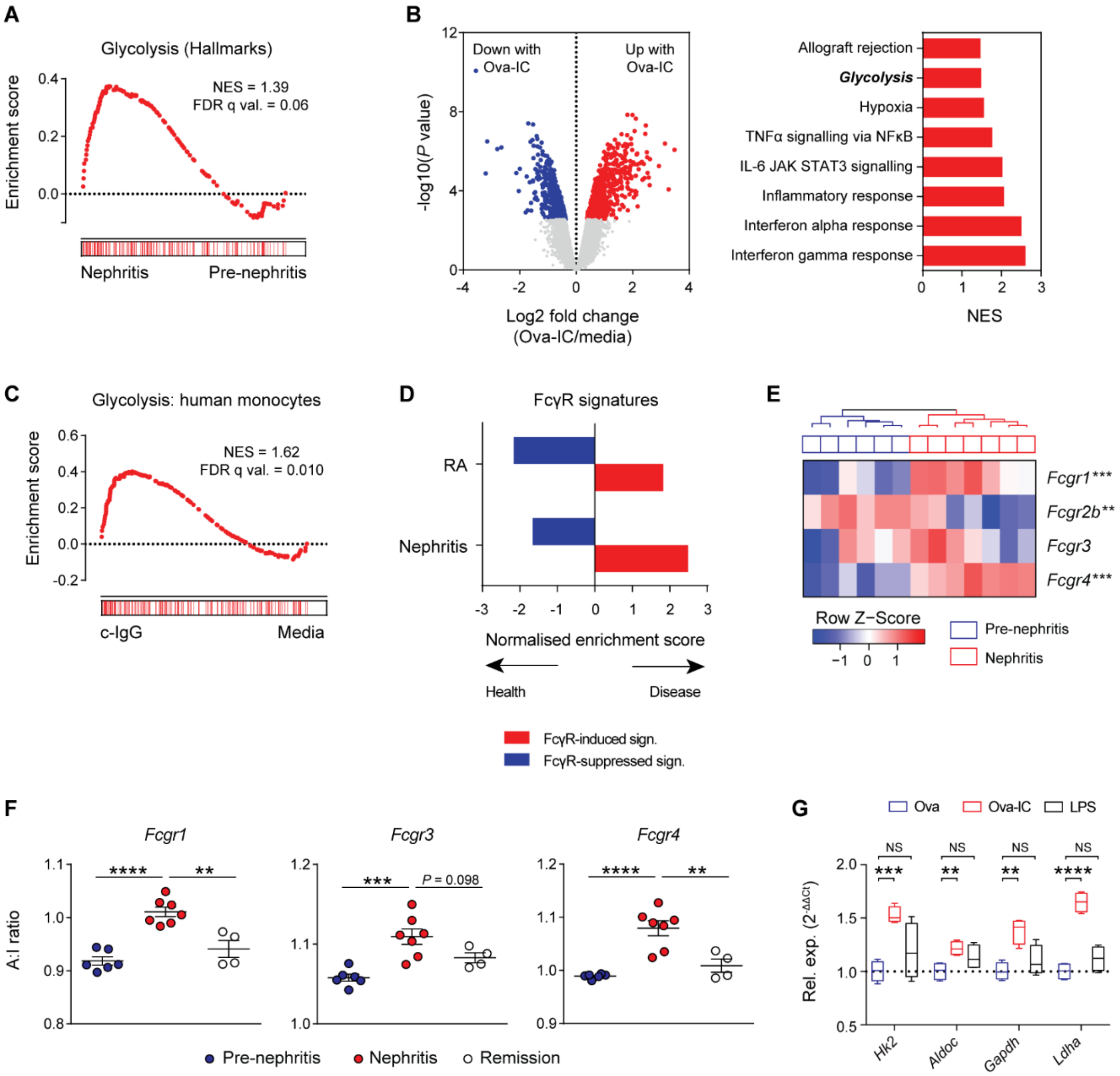

Figure S1. FcγR signalling and IgG IC signatures in macrophages. (A) GSEA of Hallmarks glycolysis pathway in renal macrophages from NZB/W mice. Data derived from GEO: GSE27045. (B) Volcano plot (left) and Hallmarks GSEA summary (right) of differentially expressed genes from murine BMDMs stimulated with Ova-IC or unstimulated for 14 h as assessed by microarray. (C) GSEA for Hallmarks glycolysis pathway in human monocytes stimulated with plate coated IgG (c-IgG). Human MDM data derived from GEO: GSE102728. (D) GSEA of IgG IC-induced (red) and suppressed (blue) transcriptional signatures in tissue macrophages from IC-associated disorders. (E) Heatmap of FcγR expression in renal F4/80^{hi} macrophages from pre-nephritis and nephritis NZB/W mice. Data derived from GEO: GSE27045. (F) FcγR mRNA A:I ratios in renal F4/80^{hi} macrophages from nephritic, pre-nephritic, and remission NZB/W mice shown in E. N = 4-7 per group. Mean ± s.e.m. are shown. (G) Expression levels of selected glycolysis genes in BMDM from *Tlr2*$^{-/-}$ *Tlr4*$^{-/-}$ mice stimulated with Ova, Ova-IC or LPS for 6 h. Data are normalized to Ova controls and *Hprt*. Mean ± s.e.m. are shown from triplicate measurements and are representative of three independent experiments. P values were calculated using limma with multiple comparisons correction using BH procedure (B, E), one-way ANOVA with multiple comparisons (F), and the two-tailed Student's t test (G). * $P < 0.05$; ** $P < 0.01$; *** $P < 0.001$; **** $P < 0.0001$.

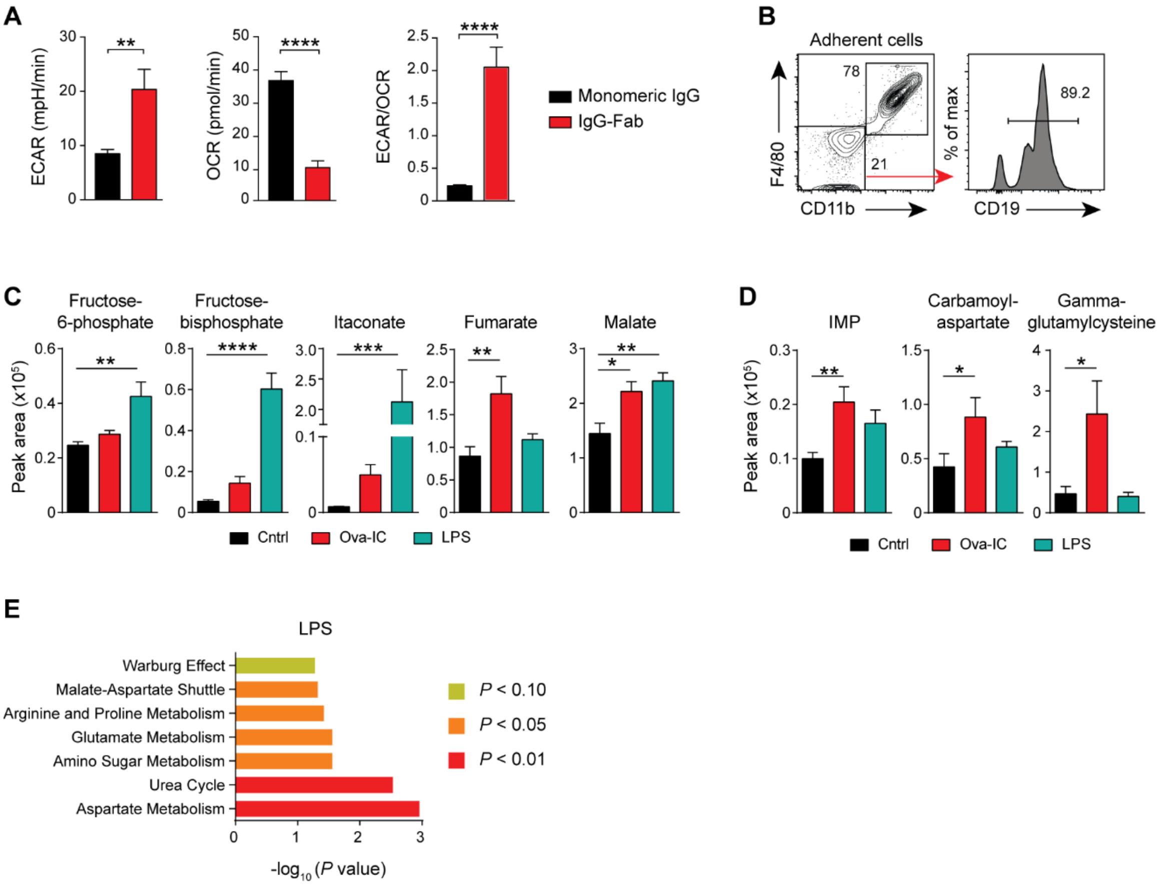

Figure S2. FcγR signalling induces a glycolytic switch in macrophages. (A) ECAR, OCR, and ECAR/OCR measured in human MDMs stimulated with monomeric IgG or IgG-Fab IC for 12 h. Mean ± s.e.m. are shown. (B) Flow cytometry of adherent peritoneal macrophages used for seahorse analysis. (C) Peak areas determined by mass spectrometry for glycolysis and TCA cycle metabolites in BMDMs stimulated with Ova-IC or LPS for 6 h. Mean ± s.e.m. are shown. *N* = 6 per group. (D) Differential metabolites between Ova-IC and LPS-stimulated BMDMs (unadjusted *P* val. < 0.05). Data are representative of two independent experiments. (E) MSEA analysis of LPS-stimulated BMDM metabolites versus control BMDMs. Data are representative of two or three independent experiments. *P* values were calculated using the two-tailed Student's *t* test (A, C, D). * *P* < 0.05; ** *P* < 0.01; *** *P* < 0.001; **** *P* < 0.0001.

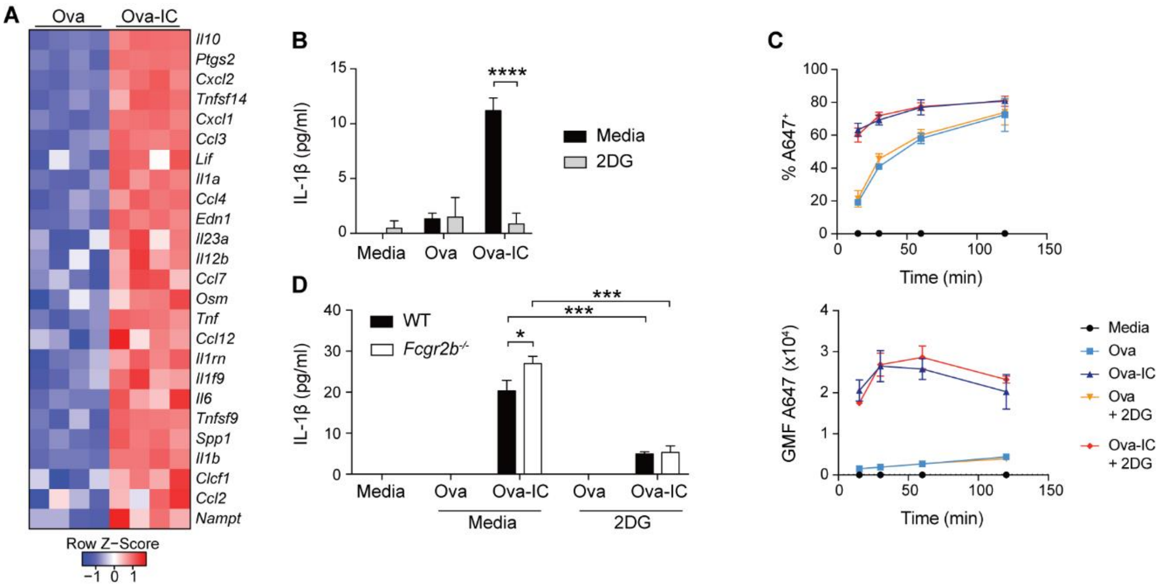

Figure S3. Glycolysis is required for IgG IC-induced IL-1β production. (A) Differentially expressed (adj. *P* value < 0.05) in colonic macrophages stimulated with Ova or Ova-IC for 4 h. Data derived from GEO: GSE109040. (B) IL-1β production by WT BMDMs stimulated with Ova, Ova-IC or unstimulated in the presence or absence of 2DG for 6 h. Mean ± s.e.m. are shown. Data are representative of three independent experiments. (C) Phagocytosis time course of Ova-A647 IC or Ova-A647 alone in BMDMs treated with 2DG as in B. Mean ± s.e.m. are shown from triplicates and are representative of two independent experiments. (D) IL-1β production by WT and *Fcgr2b*$^{-/-}$ BMDMs stimulated as in B. Mean ± s.e.m. are shown from triplicates. Data are representative of three independent experiments. *P* values were calculated using the two-tailed Student's *t* test. * *P* < 0.05; ** *P* < 0.01; *** *P* < 0.001; **** *P* < 0.0001.

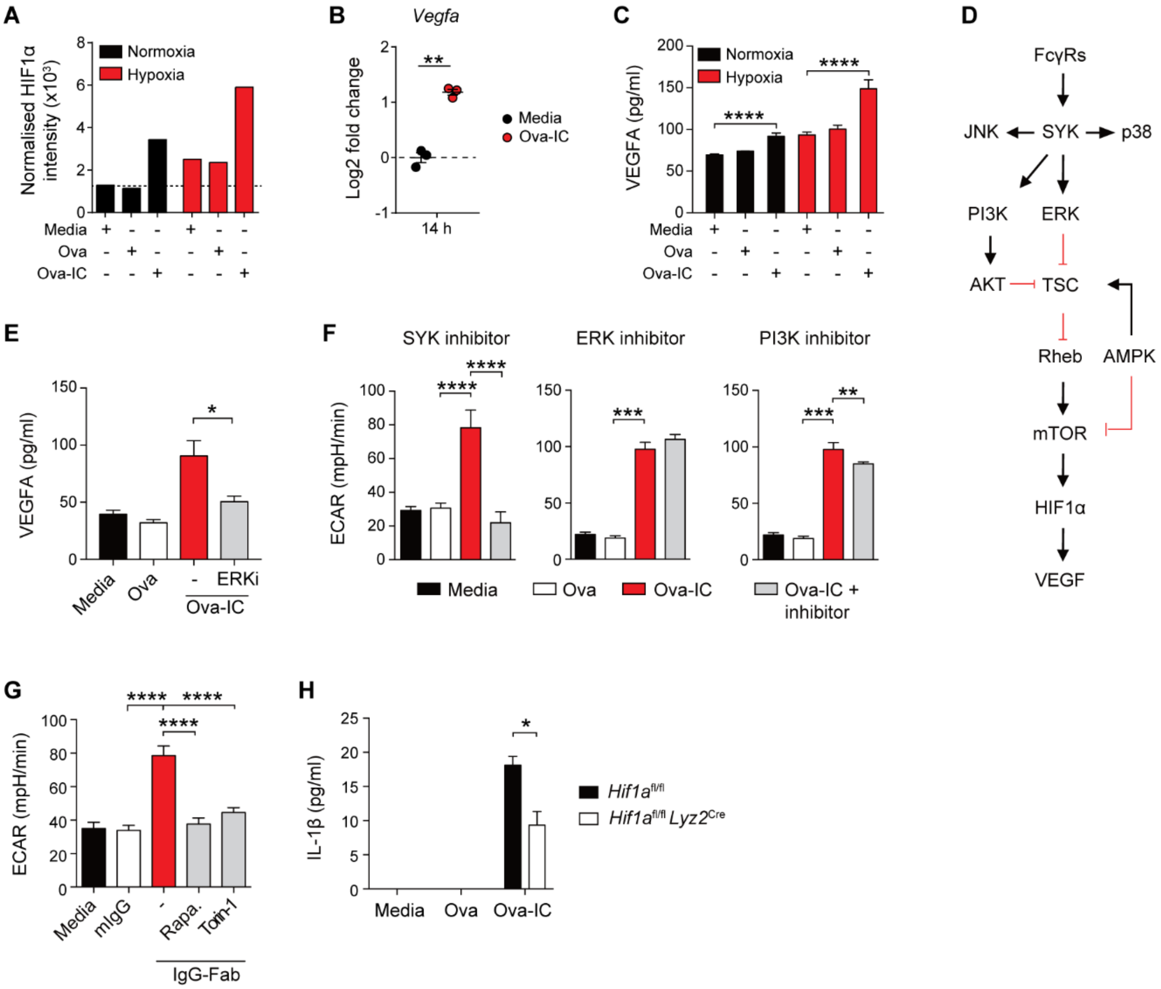

Figure S4. IgG IC induced-glycolytic switch in macrophages is mTOR-HIF1α dependent. (A) Quantification of HIF1α protein bands normalised to β-actin control bands in murine BMDMs ± Ova-IC stimulation under normoxic or hypoxic conditions. (B) *Vegfa* expression by BMDMs stimulated with Ova or Ova-IC for 14 h, determined by microarray. (C) Quantification of VEGFA production by murine BMDM stimulated with Ova, Ova-IC or control (media) under normoxic or hypoxic conditions. (D) Schematic diagram of the FcγR – SYK – mTOR – HIF1α pathway. (E) Quantification of VEGFA production by murine BMDM stimulated with Ova, Ova-IC or Ova-IC plus ERK inhibitors or unstimulated (media). BMDM were pre-treated with inhibitors (ERK inhibitor, 10 μM) for 1 h and then stimulated with IC for 20 h. (F) ECAR was measured in unstimulated (media), Ova and Ova-IC-stimulated murine BMDM with the presence of small molecule inhibitors as in E. (G) ECAR was measured in human MDM with or without the presence of mTOR inhibitors (Rapamycin, 10 nM; Torin1). MDM were pre-treated with mTOR inhibitors for 1 h and then stimulated with monomeric IgG (mIgG) or IgG-Fab IC for 20 h. (H) IL-1β production by HIF1α-deficient (*Hif1a*$^{fl/fl}$ *Lyz2*^{Cre}) and control (*Hif1a*$^{fl/fl}$) BMDM stimulated with Ova, Ova-IC or control (media) for 6 h. For all graphs, mean ± s.e.m. are shown. Data are representative of three independent experiments. *P* values were calculated using limma with multiple comparisons correction using the BH procedure (B), or the two-tailed Student's *t* test (C-H). * $P < 0.05$, ** $P < 0.01$, *** $P < 0.001$, **** $P < 0.0001$.

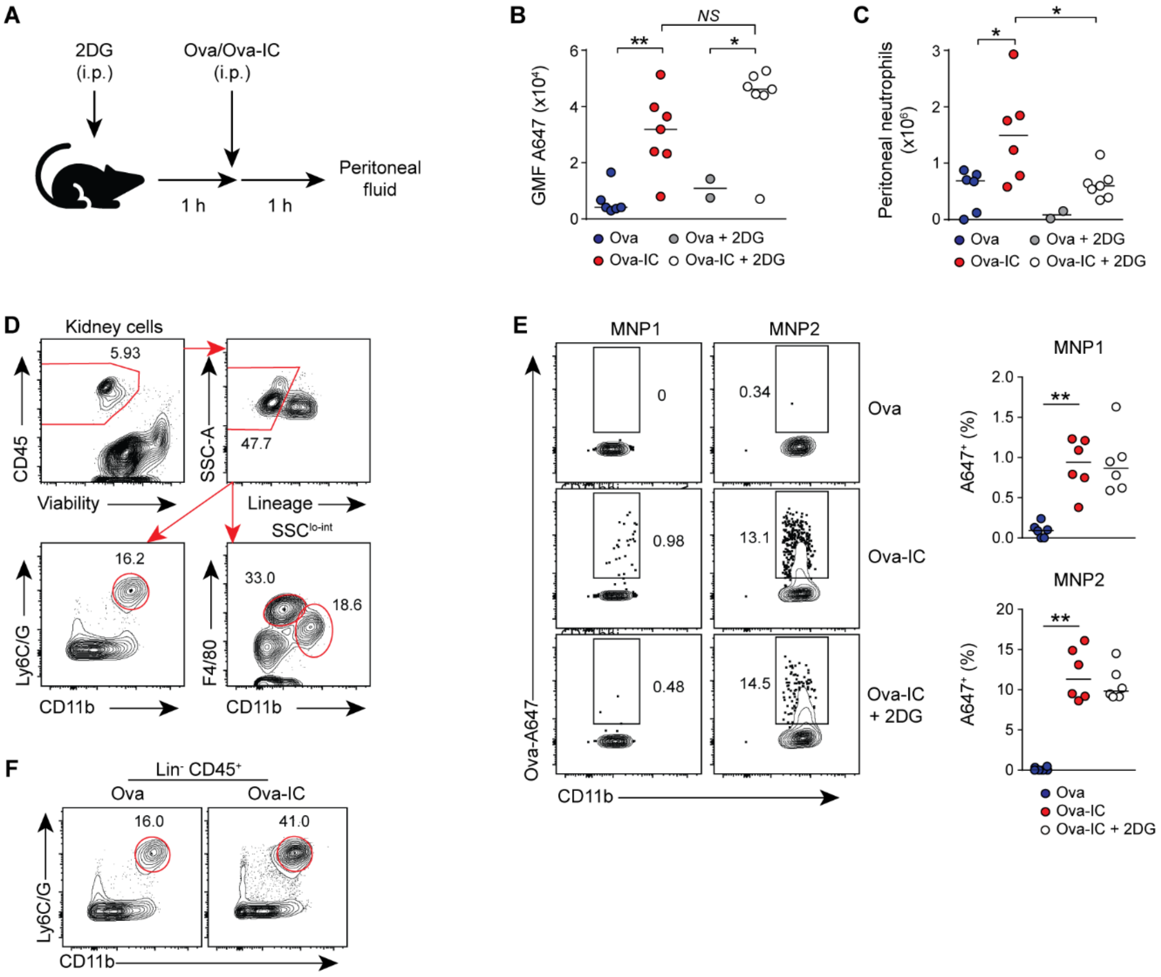

Figure S5. Inhibition of glycolysis attenuates phagocytosis of immune complexes in macrophage *in vivo*. (A) Peritoneal Ova-IC injection strategy. (B, C) Ova-IC phagocytosis by F4/80⁺ CD11b⁺ peritoneal macrophages (B) and neutrophil infiltration into the peritoneal cavity (C) following treatment as in A (*n* = 2-7 per group). Means indicated. Data representative of two independent experiments. (D) Representative flow cytometric gating strategy of kidney leukocytes by flow cytometry. (E) Representative flow cytometry plots demonstrating the uptake of A647 labelled fluorescent Ova in murine kidney MNPs following free Ova-A647, Ova-IC or Ova-IC + 2DG injection. Medians are indicated. Each datapoint represents a single kidney. Data are representative of three independent experiments. (F) Flow cytometric identification of kidney neutrophils following Ova or Ova-IC injection as in A. *P* values were calculated using the nonparametric Mann-Whitney U test (C). * $P < 0.05$; ** $P < 0.01$; *** $P < 0.001$; **** $P < 0.0001$.

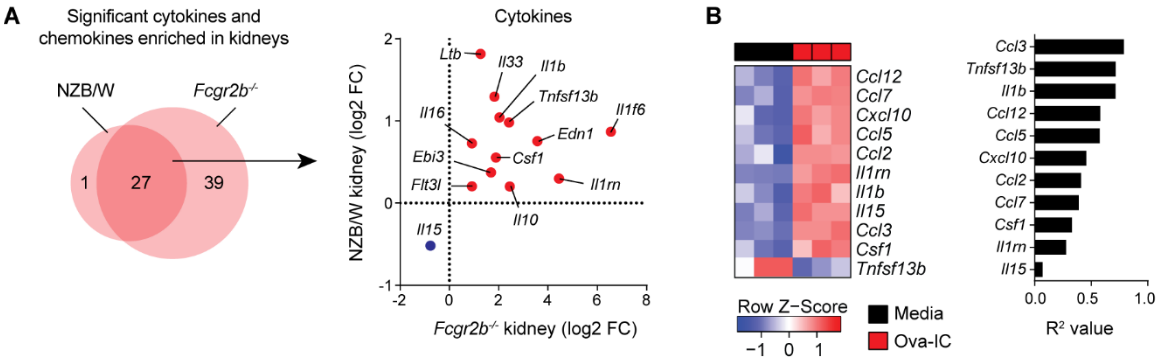

Figure S6. Inhibition of IC-induced glycolytic switch attenuates autoantibody-mediated inflammation *in vivo*. (A) Venn diagram and *xy* plot of significantly differentially expressed cytokines and chemokines within whole kidney tissue in NZB/W nephritic kidneys and *Fcgr2b*$^{-/-}$ nephritic kidneys versus controls. Data are derived from GEO: GSE27045 and GEO: GSE107705. (B) Heatmap of nephritis-associated cytokines/chemokines in BMDMs following Ova-IC stimulation (left) and correlation of these genes with *Ighg1* mRNA levels in renal tissue from NZB/W mice shown in A, *P* values were calculated using limma (NZB/W and Ova-IC microarray) or DESeq 2 method (*Fcgr2b*$^{-/-}$ RNAseq) with multiple comparisons correction using the BH procedure.

Supplementary information references

1. Lood C, *et al.* (2016) Neutrophil extracellular traps enriched in oxidized mitochondrial DNA are interferogenic and contribute to lupus-like disease. *Nat Med* 22(2):146-153.
2. Folch J, Lees M, & Sloane Stanley GH (1957) A simple method for the isolation and purification of total lipides from animal tissues. *J. Biol. Chem.* 226(1):497-509.
3. Wolf FA, Angerer P, & Theis FJ (2018) SCANPY: Large-scale single-cell gene expression data analysis. *Genome Biology* 19(1):1-5.
4. Stuart T, *et al.* (2019) Comprehensive Integration of Single-Cell Data. *Cell* 177(7):1888-1902.e1821.
5. Wolock SL, Lopez R, & Klein AM (2019) Scrublet: Computational Identification of Cell Doublets in Single-Cell Transcriptomic Data. *Cell Systems* 8(4):281-291.e289.
6. Popescu DM, *et al.* (2019) *Decoding human fetal liver haematopoiesis* pp 365-371.
7. Polański K, *et al.* (2019) BBKNN: fast batch alignment of single cell transcriptomes. *Bioinformatics* (August):1-2.
8. Traag VA, Waltman L, & van Eck NJ (2019) From Louvain to Leiden: guaranteeing well-connected communities. *Scientific Reports* 9(1):1-12.
9. Aibar S, *et al.* (2017) SCENIC: Single-cell regulatory network inference and clustering. *Nature Methods* 14(11):1083-1086.
10. Schulz C, *et al.* (2012) A lineage of myeloid cells independent of Myb and hematopoietic stem cells. *Science* 336(6077):86-90.
11. Sergushichev AA (2016) An algorithm for fast preranked gene set enrichment analysis using cumulative statistic calculation. *bioRxiv*:060012-060012.
